# Herbal medicine derived carbon dots: synthesis and applications in therapeutics, bioimaging and sensing

**DOI:** 10.1186/s12951-021-01072-3

**Published:** 2021-10-13

**Authors:** Wei-Kang Luo, Liang-Lin Zhang, Zhao-Yu Yang, Xiao-Hang Guo, Yao Wu, Wei Zhang, Jie-Kun Luo, Tao Tang, Yang Wang

**Affiliations:** 1 Institute of Integrative Medicine, Department of Integrated Chinese and Western Medicine, Xiangya Hospital Central South University, Changsha, China; 2 Hunan University of Chinese Medicine, Changsha, China; 3 The College of Integrated Traditional Chinese and Western Medicine, Hunan University of Chinese Medicine, Changsha, China

**Keywords:** Herbal medicine, Carbon dots, Synthesis, Theranostics, Medical applications, Nanomedicine

## Abstract

Since the number of raw material selections for the synthesis of carbon dots (CDs) has grown extensively, herbal medicine as a precursor receives an increasing amount of attention. Compared with other biomass precursors, CDs derived from herbal medicine (HM-CDs) have become the most recent incomer in the family of CDs. In recent ten years, a great many studies have revealed that HM-CDs tend to be good at theranostics without drug loading. However, the relevant development and research results are not systematically reviewed. Herein, the origin and history of HM-CDs are outlined, especially their functional performances in medical diagnosis and treatment. Besides, we sort out the herbal medicine precursors, and analyze the primary synthetic methods and the key characteristics. In terms of the applications of HM-CDs, medical therapeutics, ion and molecular detection, bioimaging, as well as pH sensing are summarized. Finally, we discuss the crucial challenges and future prospects.

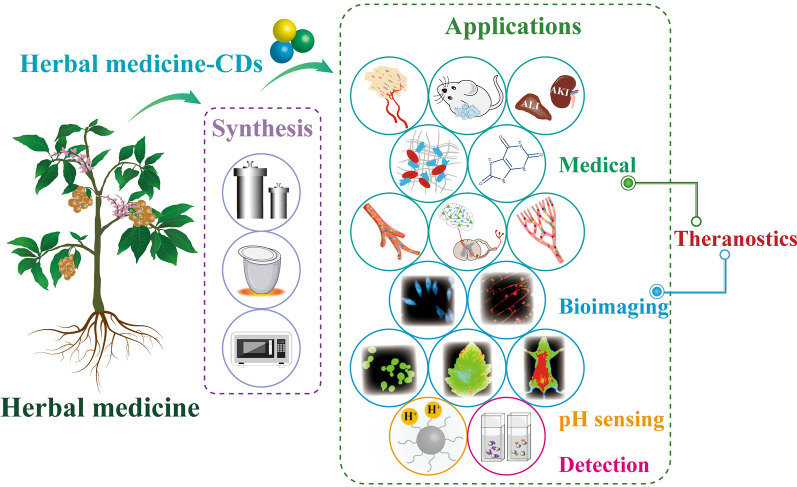

## Introduction

Initially discovered by Scrivens in early 2004, carbon dots (CDs) are emerging as a novel nanomaterial which is smaller than 10 nm in size [1]. CDs not only possess the merits from traditional semiconductors (inorganic quantum dots, etc.) and small molecules (fluorophores, etc.) but also exhibit unique properties such as photobleaching resistance, photostability, good biocompatibility and stable physicochemical characteristics [2–5]. These strengths attract much attention in various fields [6–11], especially biomedical theranostics which integrate diagnostics with therapeutics [12].

Many chemical materials (including citric acid [13], ethylene glycol [13], phenol/formaldehyde resins [14], ZnO [15], poly ethylene imine [16]and other organic solvents) are the primary precursors for CDs creation [17]. Unfortunately, these chemical-based syntheses typically involve toxic products (such as oxidative stress, reactive oxygen species (ROS), inflammation and release of metal ion) [18], that hampers clinical applications [19, 20]. To meet the challenges, scientists are devoted to searching for biomass-sourced precursors as the replaceable candidates because of their low toxicity, abundant heteroatoms, and good biocompatibility [21]. Many CDs derived from green precursors have been reviewed previously [20, 22, 23]. These reported functional green-CDs are mainly administered for anti-tumor therapy, and some were used as antimicrobial agents [5, 24–27]. Although green-CDs move a steady step on the prospective way towards biomedicine, they cannot eliminate the disadvantages of drug loading (complex manipulation and uncontrollable loading efficiency) [20].

**Fig. 1:**
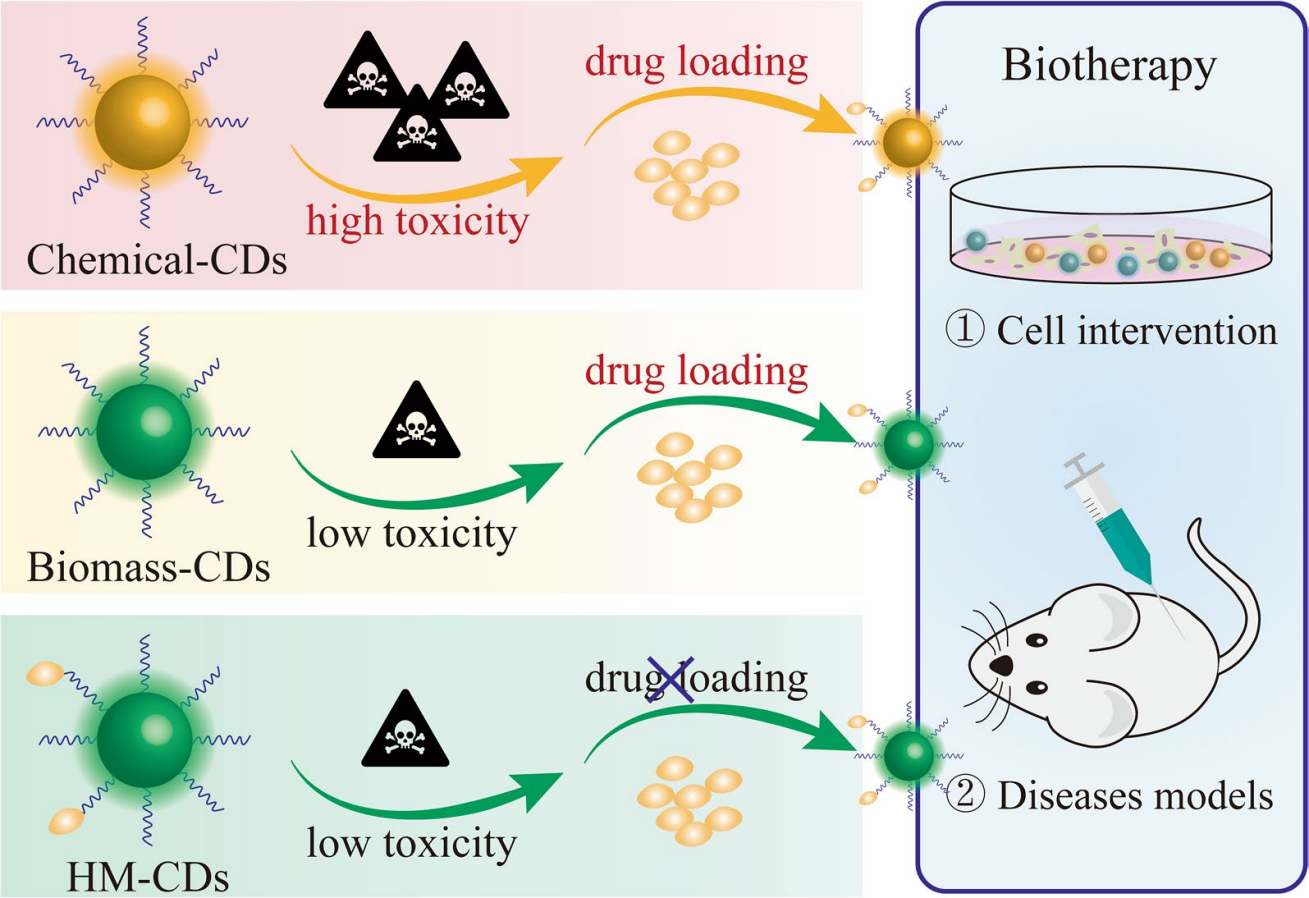
Schematic diagram of comparison of chemical-CDs, biomass-CDs and HM-CDs in biotherapy

To achieve low toxicity and avoid delivery cargoes, researchers are turning their attention to the green precursors with specific efficacy [20, 28]. Among these green precursors, herbal medicine is selected as the ideal choice. Because medicinal herbs are the natural products with huge output and approximately innocuousness. Additionally, the unique mode of diagnosis and treatment makes herbal medicine plays a key role in treating diseases [29–31]. More importantly, herbal medicine is rich in active components, thus has multiple pharmacodynamic substances [32]. (Fig. 1) These features induce herbal medicine to become a direct avenue for acquiring heteroatoms, pushing the realization of theranostics in the absence of complex delivery carries. Therefore, CDs derived from herbal medicine (HM-CDs) have attracted intensive attention in the last few years. However, no systematic discussion on the general knowledge of HM-CDs is available so far.

**Fig. 2:**
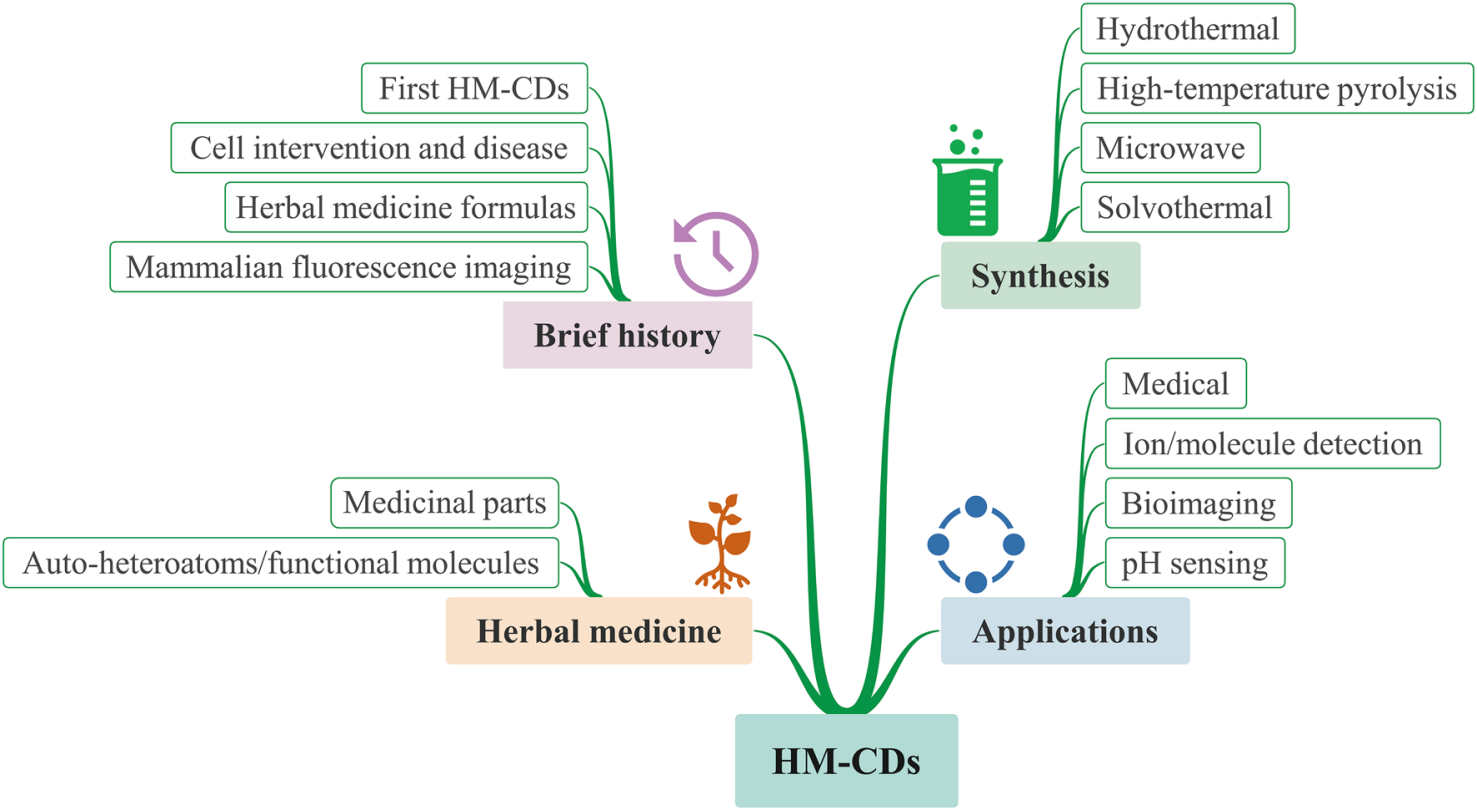
Schematic diagram of the review

In this review, we aim to highlight the merits and importance of HM-CDs. Four major sections are focused upon: (1) brief history of HM-CDs; (2) herbal medicine precursors; (3) synthetic methods; and (4) applications in biomedicine (diseases and mechanisms), ion and molecule detection, bioimaging, as well as pH sensing. Finally, we conclude with a discussion of the challenges and perspectives. The scheme of this review is clearly displayed in Fig. 2.

## A brief history of HM-CDs

**Fig. 3:**
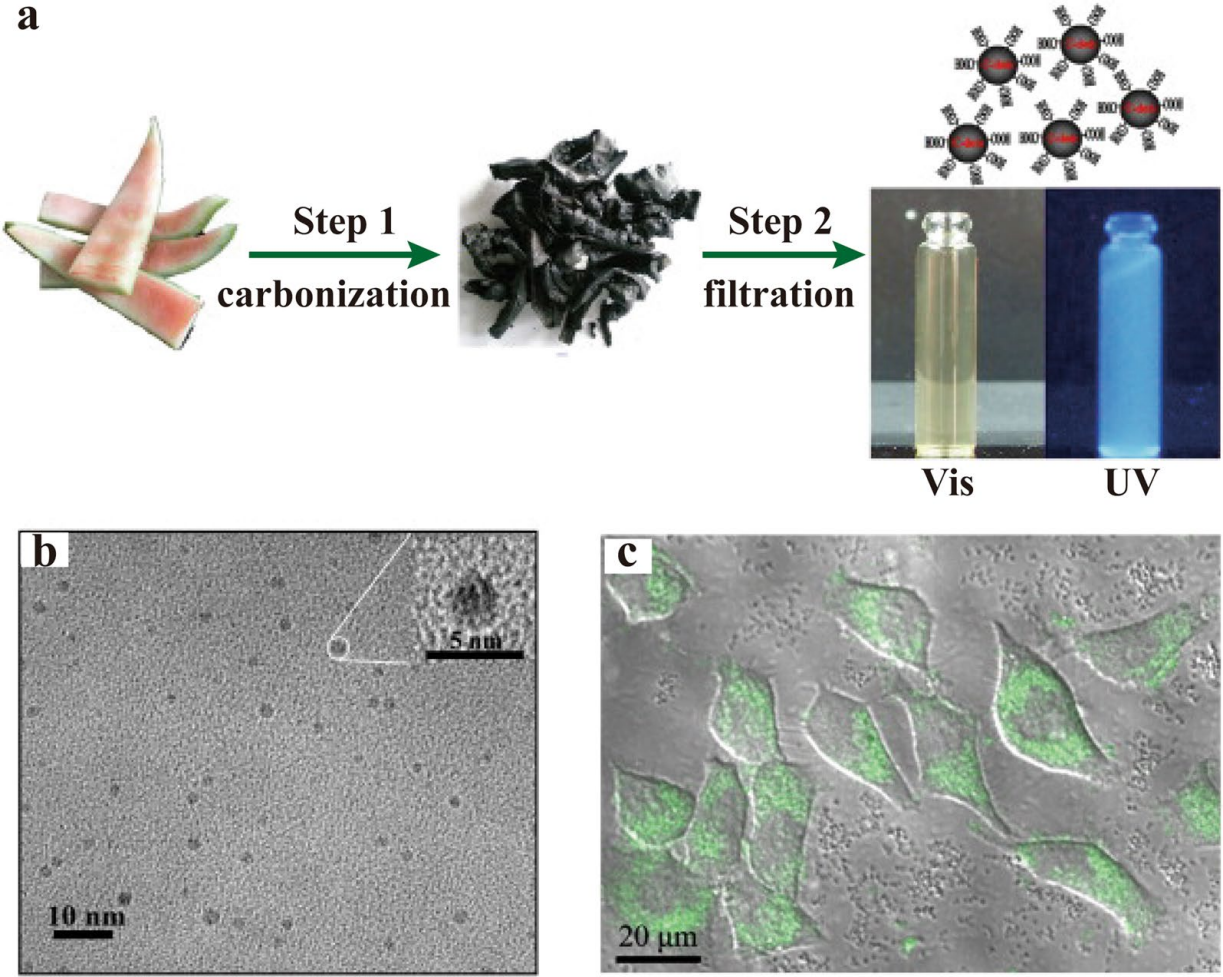
CDs derived from watermelon peels. **a** The synthesis procedure. **b** HRTEM image and the higher magnification image (inset).** c** Confocal microscopy image of Hela cells incubated with watermelon peel-CDs. Reprinted with permission from ref. [33]. Copyright (2012) Elsevier

As early as 2012, Zhou et al. [33] synthesized a water-soluble fluorescent CD using watermelon peel that is a waste and reproducible raw resource. These CDs showed strong blue luminescence without chemical oxidation and surface passivations, and they were applied in the HeLa cell imaging (Fig. 3). As an herbal medicine, watermelon peel improves fasting blood glucose and changes in hepatic metabolite accumulation [34]. The CDs derived from watermelon peel were not only the first invented HM-CDs, but also the first HM-CDs for live cell imaging. This work pioneered HM-CDs as high-performance optical imaging probes.

**Fig. 4:**
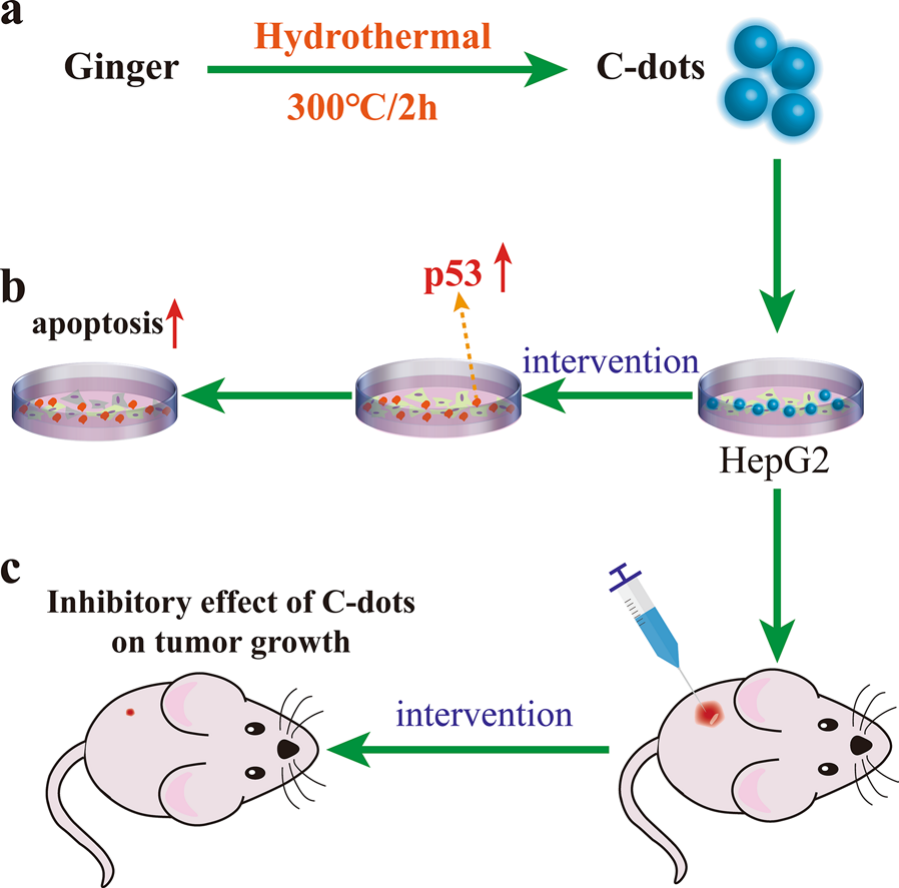
Schematic representation of the green synthesized fluorescent CDs from ginger. **a** The synthesis conditions of ginger-CDs. **b** Ginger-CDs extract selectively inhibited HepG2 proliferation.** c** Ginger-CDs can inhibit the growth of HepG2 cell-induced tumor in nude mice. Adapted from ref. [28]. Copyright (2014) Royal Society of Chemistry

In 2014, Li et al. [28] obtained the CDs prepared from ginger which is the first HM-CDs used for cell intervention and disease treatment. They found that ginger CDs without drug loading effectively inhibit human hepatocellular carcinoma cells and slower tumor growth in nude mice [28] (Fig. 4). Since then, the synthesis of CDs has initiated an age of herbal medicine-based disease treatment.

Practical compatibility is a key distinctive feature of herbal medicine [35, 36]. The effects of prescription are multi-components, multi-targets, multi-pathways and co-regulatory characters [37]. Jiaosanxian (JSX), a combination of *Fructus Crataegi* (*Jiaoshanzha*), *Fructus Hordei* Germinatus (*Jiaomaiya*) and *Massa Medicata Fermentata* (*Jiaoshenqu*), was the first herbal medicine formula for synthesizing HM-CDs. Thereupon, JSX-CDs laid milestones in the CDs derived from herbal medicine formulas. Regrettably, while this cocktail therapy is in line with the model of clinical applications of herbal medicine, JSX-CDs have more complex surface compositions and structures. Other herbal prescriptions are not used as carbon precursors so far.

**Fig. 5:**
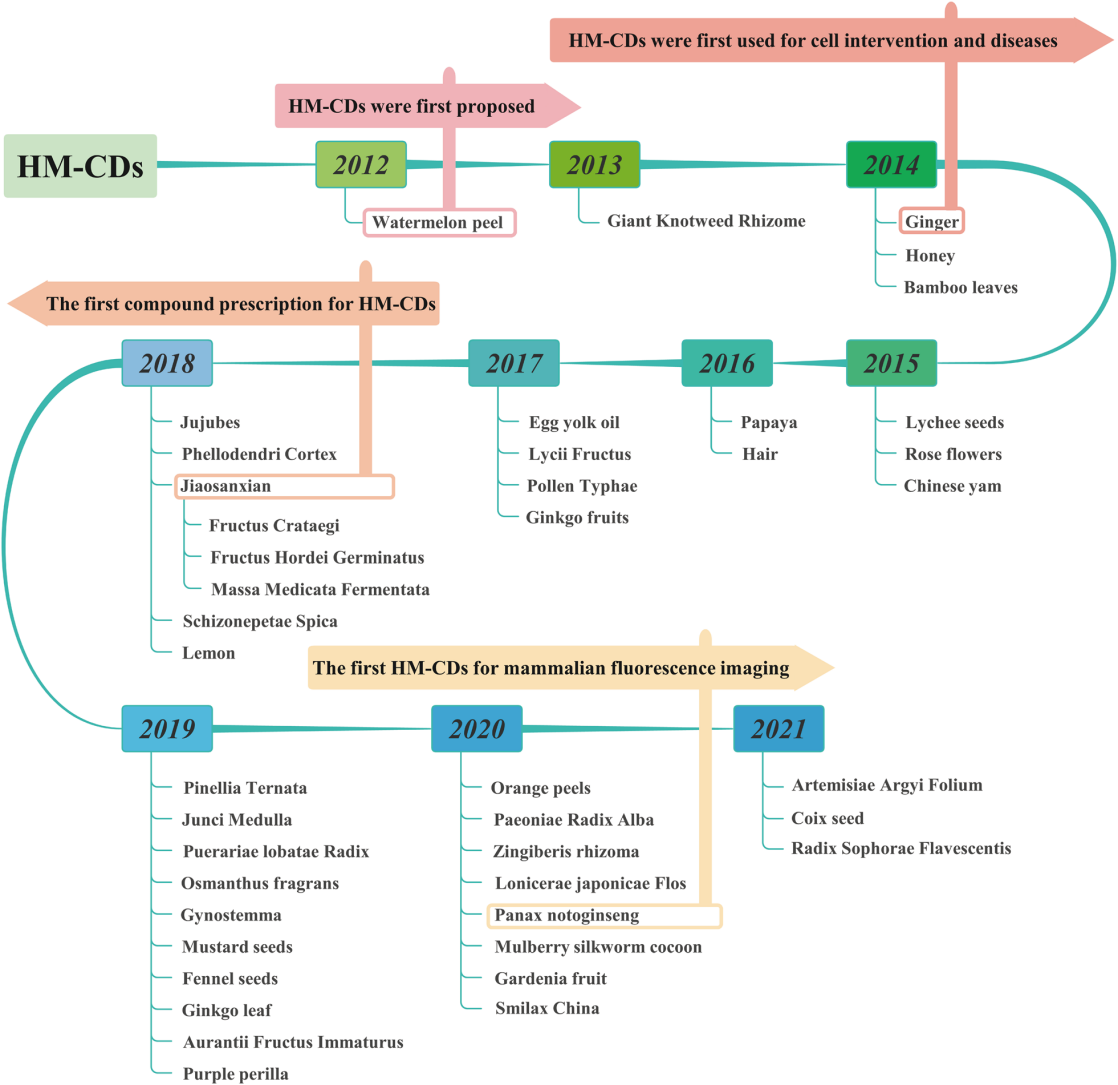
The origin and development map of HM-CDs

CDs derived from *Panax notoginseng* (*Sanqi*) for mammalian fluorescence imaging were not found until 2020 [38]. Compared with live cell imaging, in vivo live imaging tends to a clinical transformation. As of today, tens of herbal medicines have been successfully used as precursors of HM-CDs synthesis (Fig. 5).

## Herbal medicine for HM-CDs synthesis

### Medicinal parts

**Fig. 6:**
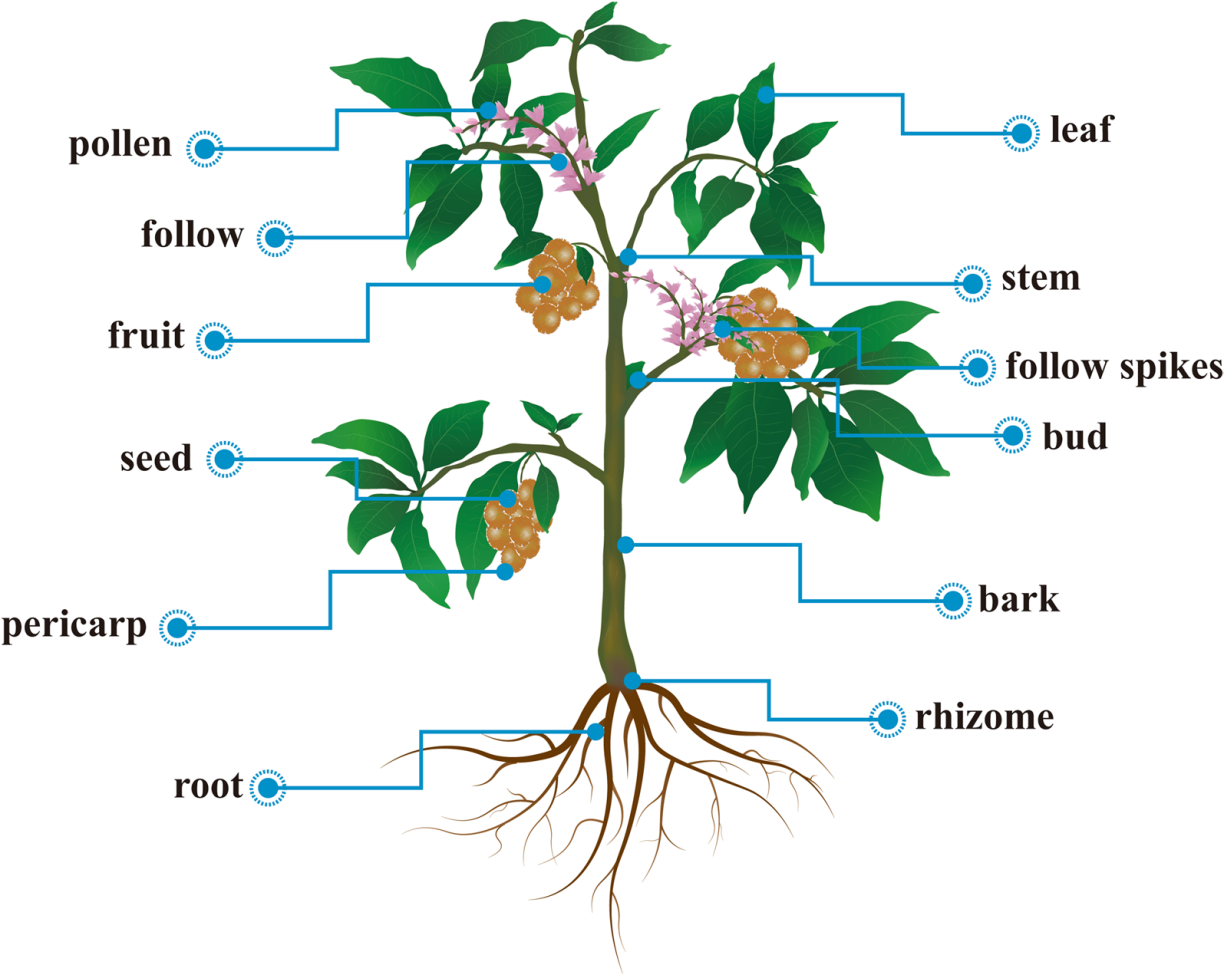
Schematic diagram of medicinal parts of herbal medicine as precursors

After analysis, all herbal precursors, we find that the overwhelming majority of all reported herbal precursors are medicinal plants including roots, flowers, leaves, fruits, seeds and other parts (Fig. 6). In herbal medicine systems, plants are also the primary therapeutic agents for treating diseases [32]. Remarkably, different medicinal parts generally contain various active components [39–41]. Since there is no detailed report on the active ingredients of HM-CDs, we are unable to elaborate on the potential relationship between HM-CD derived from different medicinal parts and their source ingredients. In addition, seldom medicines from hair [42], honey [43], egg yolk oil [44]and mulberry silkworm cocoon [45] are also used for precursors (Table 1). Future studies are urgent to explore a broader range of herbal medicine and not limited to plants.

**Table 1:** Herbal medicines for synthesizing HM-CDs

Herbal medicine	Medical parts	Refs
*Pinellia ternata*	Rhizome	[151]
*Osmanthus fragrans*	Flower	[150, 152]
Mustard seeds	Seed	[157]
Lemon juice	Fruit	[155]
Ginkgo fruits	Seed	[81]
Ginkgo leaf	Leaf	[85]
Gynostemma	Whole herb	[161]
*Puerariae lobatae* *Radix*	Root	[118]
Schizonepetae Spica	Stem/flower spikes	[97, 98]
Papaya	Fruit	[78]
*Purple perilla*	Leaf	[142]
Ginger	Rhizome	[28]
*Aurantii fructus immaturus*	Fruit	[117]
Orange peel	Pericarp	[83, 84, 103]
Lychee seeds	Seed	[96]
Rose flowers	Flower	[153]
Bamboo leaves	Leaf	[80]
Phellodendri Chinensis Cortex	Bark	[110, 127, 162]
*Radix Sophorae Flavescentis*	Root	[115]
*Lonicerae japonicae Flos*	Flower	[116]
Watermelon peel	Pericarp	[33]
Gardenia fruit	Fruit	[88]
*Artemisiae Argyi Folium*	Leaf	[134]
Pollen Typhae	Pollen	[109]
*Panax notoginseng*	Root	[38]
Hair	Tissue	[42]
Giant Knotweed Rhizome	Rhizome	[87]
Junci Medulla	Whole herb	[163]
Coix seed	Seed	[82]
Smilax China	Root	[147]
Yams	Rhizome	[79]
Jujubes	Seed	[149]
Honey	Honey	[43]
Zingiberis rhizoma	Rhizome	[135]
Fennel Seeds	Fruit	[94]
Egg yolk oil	Egg yolk	[44]
Lycii Fructus	Fruit	[148]
Paeoniae Radix Alba	Root	[126]
Jiaosanxian	Fruit/bud	[132]
Mulberry silkworm cocoon	Whole worm	[45]

### Auto-heteroatoms and functional molecules

Due to the diversity and complexity, the structures and photoluminescence mechanisms of CDs, remain poorly understood [46]. Several hypotheses have been proposed to explain the photoluminescence, such as electronic bandgap transitions of conjugated π-domains, size-dependent emission (quantum confinement effect), surface state-derived luminescence (e.g., surface defects, functional groups) and molecular luminophores, etc. [47–50]. The surface state is considered to be the primary factor [4]. Heteroatom doping (mainly N dopping [51], S dopping [52], B dopping [53], metal dopping [54]) becomes an effective way to adjust the fluorescent properties of CDs [55, 56]. The synergistic effects between various heteroatoms lead to more surface defects, reduce nonradiative recombination, thus improve their fluorescence intensity (e.g., B, S, N doped-CDs [57], N, P doped-CDs [58]and N, S doped-CDs [59]). Briefly speaking, a growing appreciation that precursors with abundant heteroatoms may avoid the additional doping.

**Fig. 7:**
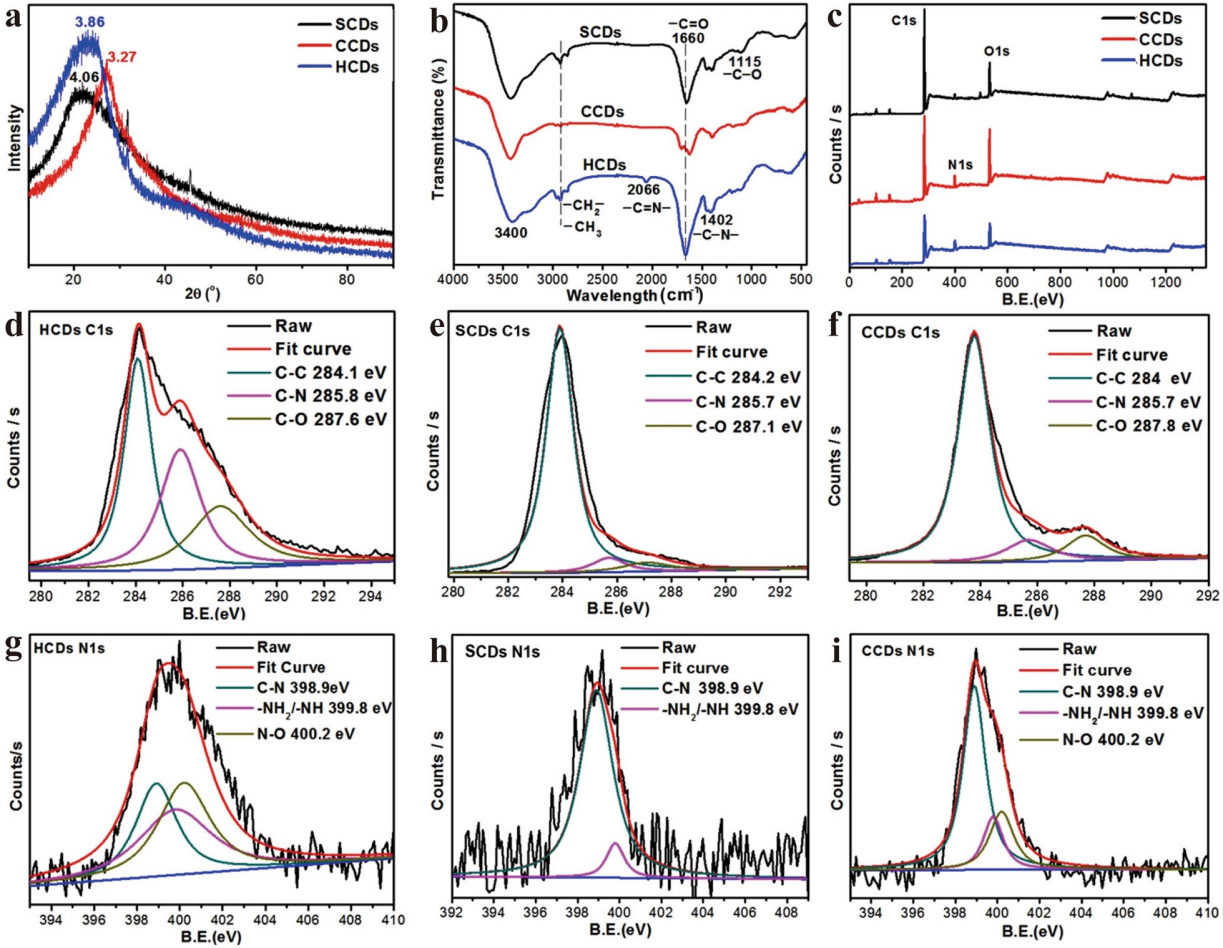
Characterization of chemical structure of hair-CDs (HCDs), skin-CDs (SCDs) and citric acid-CDs (CCDs). **a** XRD patterns,** b** Fourier transform infrared spectra, **c** XPS spectra,** d** C1s profile of HCDs, **e** C1s profile of SCDs,** f** C1s profile of CCDs, **g** N1s profile of HCDs,** h** N1s profile of SCDs,** i** N1s profile of CCDs. Reprinted with permission from ref. [42]. Copyright (2016) Springer Nature

As we all know, natural sources consist of organic molecules which tend to serve as carbon precursors or functional groups [60]. Multifarious herbal medicines are rich in biological activities from polysaccharide [61], proteins [62], nucleic acids [63] and phospholipid [42], etc. These activities are the potent sources of functional groups (e.g., C, H, N and O) without extra surface passivation or doping. For example, the hair-derived CDs are the only HM-CDs that use human derivatives as a precursor [42]. The abundant microelements (e.g., phosphorus in phospholipid and sulfur in amino acid) tuned the intrinsic properties, enhancing the photoluminescence [42]. The nitrogen contents of hair CDs and skin CDs are higher than of citric acid CDs (Fig. 7). Besides, the protein is a biological macromolecule mainly includes chemical elements, such as carbon, hydrogen, oxygen and nitrogen. In light of the above discussions, these elements may contribute to the performance improvement of CDs. Therefore, the protein-rich animal drugs are expected to be an optimal precursor of HM-CDs synthesis.

## Synthetic methods of HM-CDs

The methods of CDs synthesis fall into two categories: top-down and bottom-up strategies [64–67]. The former refers to large particles broken by physical, chemical or electrochemical technologies [23, 68], while the latter means nanoparticles formed by small molecules [23, 69, 70]. By contrast, bottom-up methods are more popular with scientists, because this technology shows double advantage of being straightforward and economical [71]. Bottom-up technique involves hydrothermal method [72], high-temperature pyrolysis method [73], solvothermal method [74]and microwave-based method [75]. Of these methods, hydrothermal synthesis is considered the greenest way to prepare HM-CDs [23, 76] (Table 2).

**Table 2:** Synthesis of HM-CDs

Sources	Synthetic methods	Reaction temperature (℃/W)	Reaction time	QY (%)	Average particle size (nm)	Particle size distribution (nm)	Refs
Pinellia Ternata	Hydrothermal	180	9 h	21.30	5.2	5.0–6.0	[151]
*Osmanthus fragrans*	Hydrothermal	180	10 h	21.90	3.1	2.0–6.0	[152]
*Osmanthus fragrans*	Hydrothermal	240	5 h	18.53	2.23	1.0–3.5	[150]
Mustard seeds	Hydrothermal	180	4 h	4.60	4.58 ± 0.26	2.0–8.0	[157]
Lemon juice	Hydrothermal	120	3 h	16.70	3.1	2.0–4.5	[155]
Ginkgo fruits	Hydrothermal	160/180/200	8/12/16 h	3.33	3.81	2.0–6.0	[81]
Ginkgo leaf	Hydrothermal	200	10 h	22.80	3	2.0–4.0	[85]
Papaya	Hydrothermal	200	5 h	18.98	3.4	2.0–6.0	[78]
*Purple perilla*	Hydrothermal	260	5 h	9.01	2.8	–	[142]
Orange peel	Hydrothermal	200	4 h	18.57	2.34	–	[84]
Orange peel	Hydrothermal	200	6 h	11.37	2.9 ± 0.5	–	[83]
Bamboo leaves	Hydrothermal	200	6 h	7.10	3.6	3.4–4.2	[80]
*Panax notoginseng*	Hydrothermal	180	12 h	8.54	7.8	7.2–8.4	[38]
*Panax notoginseng*	Hydrothermal	180	12 h	18.41	6.66	5.58–7.75	[38]
Giant Knotweed Rhizome	Hydrothermal	200	3 h	11.50	35	–	[87]
Jujubes	Hydrothermal	190	6 h	–	3.12	–	[149]
Honey	Hydrothermal	100	2 h	–	–	Mainly 2 nm	[43]
Lycii Fructus	Hydrothermal	180	5 h	17.20	3.3	2.0–5.0	[148]
Ginger	Hydrothermal	300	2 h	13.40	4.3 ± 0.8	–	[28]
Yams	Hydrothermal	200	2 h	–	–	1.5–4.0	[79]
Gardenia fruit	Hydrothermal	180	5 h	10.70	2.08	1.2–3.5	[88]
Coix seed	Hydrothermal	180	3 h	17.43	2.4 ± 0.2	2.1–2.7	[82]
Smilax China	Hydrothermal	200	5 h	22.37	2.10 ± 0.29	0.5–3.0	[147]
Puerariae lobatae Radix	High-temperature pyrolysis	300	1 h	3.20	–	3.0–10.0	[118]
Schizonepetae Spica	High-temperature pyrolysis	350	1 h	6.31	–	1.29–6.87	[98]
Schizonepetae Spica	High-temperature pyrolysis	350	1 h	2.26	–	0.8–4.0	[97]
*Aurantii fructus immaturus*	High-temperature pyrolysis	350	1 h	7.20	–	1.1–4.4	[117]
Lychee seeds	High-temperature pyrolysis	300	2 h	10.60	1.12	0.4–2.0	[96]
Phellodendri Chinensis Cortex	High-temperature pyrolysis	350	1 h	–	2.84 ± 0.89	–	[127]
Phellodendri Chinensis Cortex	High-temperature pyrolysis	350	1 h	9.62	–	1.2–4.8	[110]
Phellodendri Chinensis Cortex	High-temperature pyrolysis	400	1 h	5.63	1.93 ± 0.53	0.5–3.6	[162]
*Radix Sophorae Flavescentis*	High-temperature pyrolysis	350	1 h	1.08	–	Mainly 2.0–3.0	[115]
*Lonicerae japonicae* Flos	High-temperature pyrolysis	350	1 h	0.50	–	1.0–10.0	[116]
Watermelon peel	High-temperature pyrolysis	220	2 h	7.10	2.0 ± 0.5	–	[33]
*Artemisiae Argyi Folium*	High-temperature pyrolysis	350	1 h	0.19	–	6.0–10.0	[134]
Pollen Typhae	High-temperature pyrolysis	350	1 h	–	4.2 ± 1.4	2.0–8.0	[109]
Junci Medulla	High-temperature pyrolysis	350	1 h	0.12	–	1.0–8.0	[163]
*Zingiberis rhizoma*	High-temperature pyrolysis	350	1 h	5.20	3.00 ± 0.77	2.23–3.77	[135]
Fennel Seeds	High-temperature pyrolysis	500	3 h	9.50	3.90 ± 0.91	–	[94]
Egg yolk oil	High-temperature pyrolysis	260	1 h	5.01	–	Mainly less than 10 nm	[44]
Paeoniae Radix Alba	High-temperature pyrolysis	350	1 h	5.34	–	1.0–2.4	[126]
Jiaosanxian	High-temperature pyrolysis	–	–	7.95	–	4.4–6.4	[132]
Mulberry silkworm cocoon	High-temperature pyrolysis	350	1 h	6.32	–	2.26–9.35	[45]
Gynostemma	High-temperature pyrolysis	400	4 h	5.7	2.49 ± 0.43	–	[161]
Hair	High-temperature pyrolysis/microwave	300/400	2 h/4 min	86.06	3.57	2.0–6.0	[42]
Rose flowers	Microwave	–	–	–	–	4.0–6.0	[153]
Ginkgo fruits	Microwave	800	5/10/15 min	0.65	2.82	2.0–4.0	[81]
Orange peel	Microwave	900	1 min	16.20	4.2	3.0–5.0	[103]
Papaya	Solvothermal	200	5 h	18.39	10.8	8.0–18.0	[78]

### Hydrothermal method

#### Synthesis procedure

**Fig. 8:**
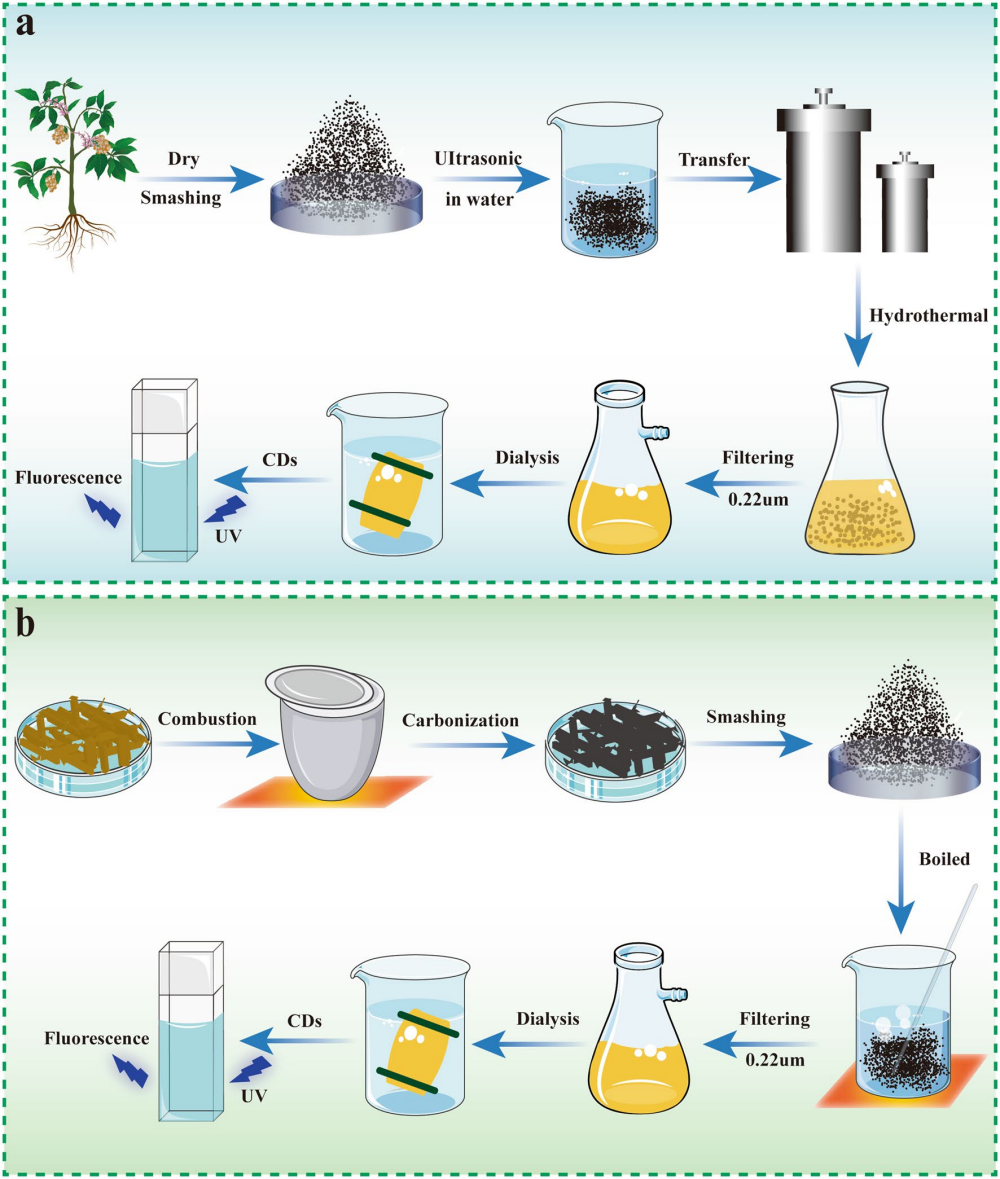
Schematic diagram on the synthesis process of HM-CDs. **a** The hydrothermal synthesis process of HM-CDs, **b** High-temperature pyrolysis process of HM-CDs

Hydrothermal synthesis is green without adding organic matter [77], that is the primary reason for preparing the CDs derived from natural substances. The surface of CDs does not require additional passivation to maximize safety and minimize toxicity. Before preparation, dried herbs are cut into small pieces or powder in ultrapure water. After sonication, the mixture is transferred to a Teflon-lined stainless steel autoclave and heated at a specific temperature. To obtain pure CDs, the suspension needs further filtration with a 0.22 μm cellulose filtration membrane and dialyzation with a dialysis bag for several days. (Fig. 8a).

#### Reaction temperature and time

Reaction temperature and time can affect the performance of HM-CDs. The hydrothermal reaction temperature of HM-CDs (such as CDs derived from papaya [78], yams [79], bamboo leaves [80]) was normally 100–200 ℃. Li et al. [81] synthesized the nitrogen-doped CDs from ginkgo fruits (H-N-CDs) at different temperatures and times. These CDs had the best fluorescence intensity, maximum quantum yield (QY) and lifetime (from the excited state to the ground state) when the temperature was set at 200 ℃. Another study [43] produced the honey-CDs at 100 ℃ for 2 h. However, the CDs merely was stored steadily for 3 months at 4 ℃. The difference between the fluorescence intensity of honey-CDs was no longer significant when the synthesis time was extended to 12 h and even 16 h, suggesting that the fluorescence intensity may have a saturated state. This phenomenon was also observed in coix seed CDs [82]. Specifically, the fluorescence intensity of coix seed-CDs decreased with the temperature rose from 15 to 80 °C [82]. The reason for this is that there exists the increased molecular collision frequency, non-radiative transition rate, as well as the constant radiation transition rate at high temperature [82]. Therefore, the CDs can function as a temperature sensor. The third representative research reported CDs derived from orange peel at different time points under the premise of the same temperature. This study noted that the QY decreased with the extension of reaction time, and the diameter increased slightly concurrently [83, 84].

#### HM-CDs derived from different medicinal parts and varieties

CDs derived from the same part of different herbal species have distinct properties. The most typical study is that the scientists developed CDs derived from 14 different strains of orange peels. Different varieties gave rise to significant differences in QY under the same preparation conditions [84]. This study further highlights that the QY may be associated with the amounts of volatile oils, which would drive the research of HM-CDs derived from the pericarp.

What’s more, CDs extracted from different parts of the same herbal medicine also possess divergent performances. For instance, two HM-CDs derived from different parts of the same ginkgo tree were discovered [81, 85]. The research team [85] prepared ginkgo leaves-CDs with higher QY (22.80%) using hydrothermal synthesis. In contrast, the QY of ginkgo fruits-CDs was only 3.33% in the same synthetic way [81]. The evidence demonstrates that herbal medicine from various parts of the same plant leads to considerable discrepancies of HM-CDs, that may be due to the differential components.

#### Size and blood–brain barrier permeability

Biological barriers, such as the blood–brain barrier (BBB), hinder the infiltration of herbal macromolecules [86]. To overcome this problem, the world pays more attention to HM-CDs whose size is nanometer scale. In the hydrothermal synthesis, except for *Giant Knotweed Rhizome*-CDs [87], the average HM-CDs particle size was less than 10 nm (Table 2). The minimal average diameter was 2.08 nm [88]. The nanoscale HM-CDs prominently ameliorate the permeability, exerting more strengths than herbal medicine. The BBB penetration mechanism is classified into active transport and passive transport [89]. In the HM-CDs, one report noted that Pn-CDs could cross the BBB, which may be due to the ultra-small size, abundant surface functional groups, and the strong affinity to endothelial cell membrane of BBB [38]. But the molecular uptake mechanisms of Pn-CDs should be further evaluated. Furthermore, the CDs are propitious to the delivery of macromolecules via the carrier-mediated transport by covalently binding with drugs. Thus, these features enable the CDs improve the BBB permeability through passive transport [90]. Ashrafizadeh et al. [26] summarized the novel neuro-drug delivery systems for various neurological disorders using functionalized CDs as carriers. However, expensive ligands for modification limit their broad applications [26]. Herbal medicine is prescribed chronically for neurological disorders [91]. As a result, HM-CDs may enhance the BBB permeability of some macromolecules under non-functionalized conditions. It has the potential to be a novel tactic for herbal medicine to overcome biological barriers.

### High-temperature pyrolysis method

#### Synthesis procedure

High-temperature pyrolysis is a more common method in addition to hydrothermal synthesis. The organic substances in the precursors can be gradually converted into CDs via heating, dehydration, degradation and carbonization under high temperature in either vacuum or inert atmospheres throughout synthesis [92]. The process is facile, solvent-free, low-cost, and amenable to large-scale production [93]. Herbal medicine is first put into the crucible and heated at a specific temperature with the muffle furnace until it was carbonized. The charred medicine is then crushed and boiled in ultrapure water, and the upper liquid is collected. After filtration through a 0.22 μm microporous membrane, the solution is dialyzed using a dialysis bag for several days to harvast the purified CDs (Fig. 8b).

#### Comparison of high-temperature pyrolysis and hydrothermal methods

**Fig. 9:**
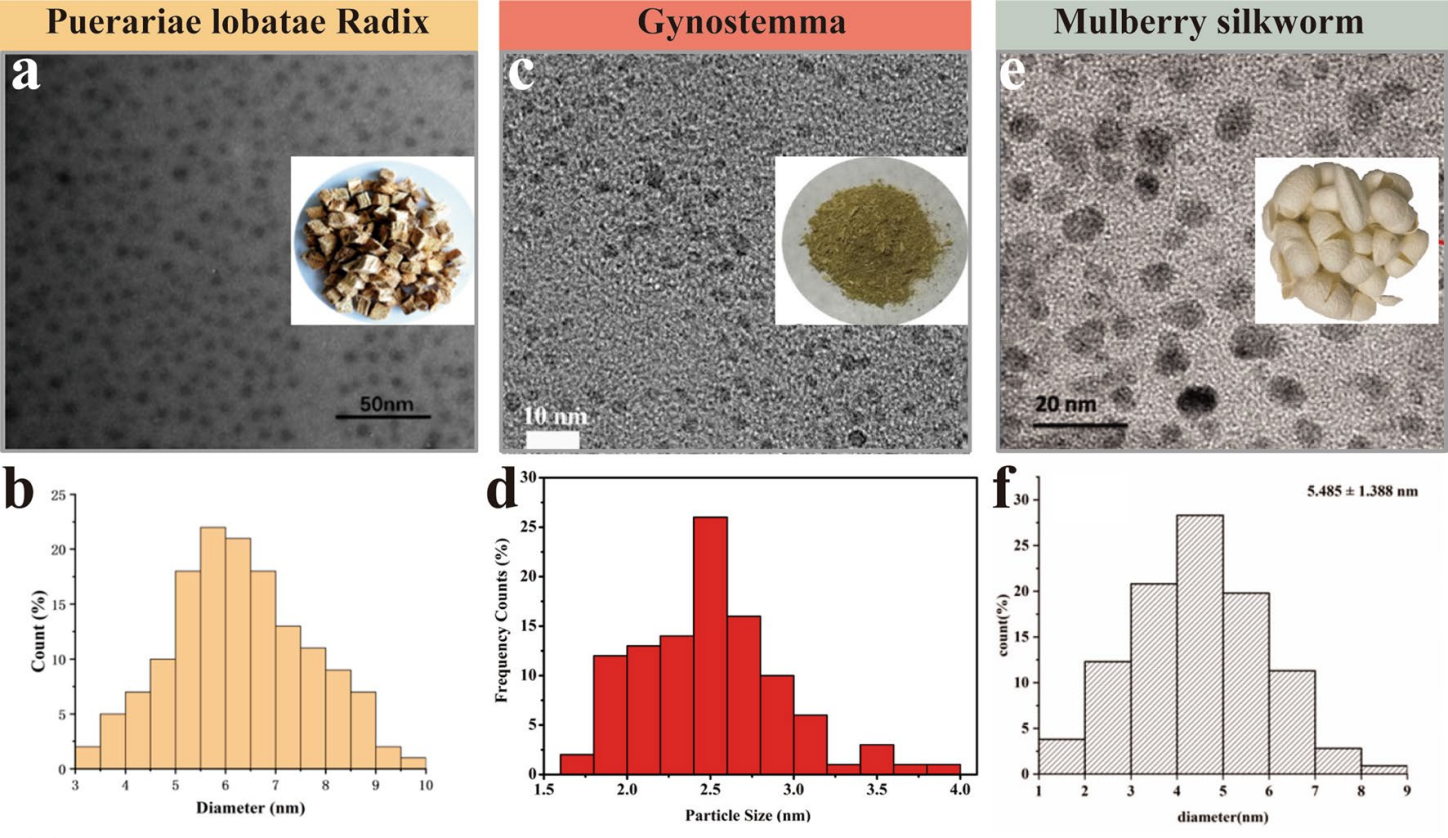
Representative transmission electron microscopy (TEM) images of HM-CDs prepared by high-temperature pyrolysis. **a** HM-CDs are derived from *Puerariae lobatae Radix* (inset),** b** TEM size distribution of *Puerariae lobatae Radix*-CDs, **c** HM-CDs are derived from Gynostemma (inset), **d** TEM size distribution of Gynostemma-CDs, **e** HM-CDs are derived from the mulberry silkworm cocoon (inset), **f** TEM size distribution of mulberry silkworm-CDs. Reprinted with permission from ref. [45, 118, 161]. Copyright (2019) by the authors. Licensee MDPI, Basel, Switzerland. Copyright (2019) American Chemical Society. Copyright (2019) The Authors. Published by Informa UK Limited

Compared with hydrothermal synthesis, high-temperature pyrolysis usually requires a higher reaction temperature (around 300 ℃). Concomitantly, the reaction temperature is higher, the heating time shorter (Table 2). Dager et al. [94] prepared a mono-dispersed CDs using the fennel seeds at a constant temperature of 500 °C for 3 h. These CDs were stored for up to 15 months and had excellent colloidal solubility, photostability and environmental stability. In the existing HM-CDs prepared by the high-temperature pyrolysis, the minimum heating temperature was 220 °C. Blue-light CDs were prepared with watermelon peel as a carbon source at this temperature and dissolved in several solvents [33]. Another vital issue is the particle size of HM-CDs synthesized by both of the methods. While studies pointed out that the hydrothermal synthesis was easier to achieve the narrow size distribution of CDs than the pyrolysis [95], the latter can also achieve a smaller size. After checking statistical literature, we find that the diameters of HM-CDs prepared by pyrolysis are about 5 nm under the existing synthetic conditions (Fig. 9). It seemingly represents no noticeable difference in the particle diameters of HM-CDs synthesized by the two approaches.

#### Quantum yield and particle size

Unlike the previous review [93], QY of pyrolysis synthesis is lower than that of hydrothermal because of the diversity of carbon sources. Except for CDs with a high QY (10.60%) synthesized from lychee seeds [96], the average QY of others was less than 10% (Table 2). Remarkably, as for the synthesis of *Schizonepetae Herba Carbonisata*-CDs (SHC-CDs), two prior studies yielded divergent results under a uniform condition. Both of Zhang et al. [97] and Sun et al. [98] created SHC-CDs. The former reported a SHC-CDs with an average size of 0.8–4.0 nm and a QY of 2.26%. While in the latter publication, the average size and QY of SHC-CDs were 1.29–6.87 nm and 6.31%, respectively. These findings illustrate the destabilization of this approach. Optimal synthesis continues to be explored. By combining pyrolysis with microwave methods, Zhang and others [42] produced hair CDs with high QY (86.06%), much higher than that of citric acid CDs (19.73%). Except for differences in carbon sources, the combination of both synthetic strategies may offer potential benefits. They also fabricated skin CDs with higher QY (51.35%), demonstrating that protein-rich materials are more suitable as precursors [42]. Hence, animal-derived herbal medicine may be the most promising drugs for HM-CDs synthesis with high QY in the future.

### Microwave method

#### Synthesis procedure

The synthesis process of microwave is similar to that of pyrolysis. Differently, the former can reach the intended energy in a short time and ensure that the precursors are heated evenly. Moreover, it’s an electromagnetic wave (wavelength range between 1 mm and 1 m), which arises the cleavage of chemical bonds via delivering energy [99]. Thus, it enables us to fabricate uniform CDs by breaking chemical bonds. This method significantly reduces the response time and enhances the effectiveness of preparation [75, 100, 101]. (Table 2) Additionally, microwave-assisted hydrothermal synthesis has been reported as an alternative to traditional hydrothermal synthesis [102].

#### Comparison with other methods

To compare with other methods, Li et al. [81] prepared two ginkgo fruits-CDs (H-CDs/M-CDs) using hydrothermal (H) and microwave (M) methods, respectively. The synthesis of M-CDs took only 5–15 min, much shorter than that of hydrothermal method. The particle size was relatively smaller (Table 2). However, the fluorescence property of H-CDs was much better than that of M-CDs. On one hand, it is because of H-CDs with more regular and uniform morphology. On the other hand, the luminescence mechanism plays an important role. The QY and lifetime of H-CDs were greater than M-CDs and had a more vigorous fluorescence intensity (Table 2). Notably, the microwave method could even prepare CDs derived from orange peel within 1 min and yield up to 16.20% [103]. These CDs had high green fluorescence with excitation-dependent emission fluorescence behavior. Indubitably, microwave synthesis may be better than the hydrothermal and pyrolysis methods according to reflection time and efficiency. Although the microwave method has the above positives, it remains the rare application for HM-CDs synthesis.

### Solvothermal method

Unlike hydrothermal synthesis, the solvothermal method involves a variety of solvents other than water [54, 104]. Wang et al. [78] prepared ethanol-papaya CDs (E-CDs) and water-papaya CDs (W-CDs), respectively. There were a vast number of saccharides and a small number of water-soluble macromolecules in the water-based medium, all of which were beneficial to the production of W-CDs. Conversely, more organic macromolecules in 90% ethanol led to E-CDs with larger size [78]. As a result, the W-CDs had better fluorescence stability, exposing the ethanol defects.

Two most commonly methods are hydrothermal and high-temperature pyrolysis. While hydrothermal synthesis is called the greenest manner, the reaction time is relatively long. This drawback causes inefficiency of synthesis. Other methods are rarely selected to prepare HM-CDs currently, but have been pervasively used to synthesize CDs derived from other precursors, especially microwave. Short reaction time, high efficiency, heat uniformity and other characteristics will make the microwave method ideal for HM-CDs synthesis.

Collectively, the particle size of HM-CDs obtained by all methods were unevenly distributed. QY was generally low. The defects essentially hinder the utility in biomedicine and the realization of future commercialization.

## Applications of HM-CDs

### Medical applications

**Fig. 10:**
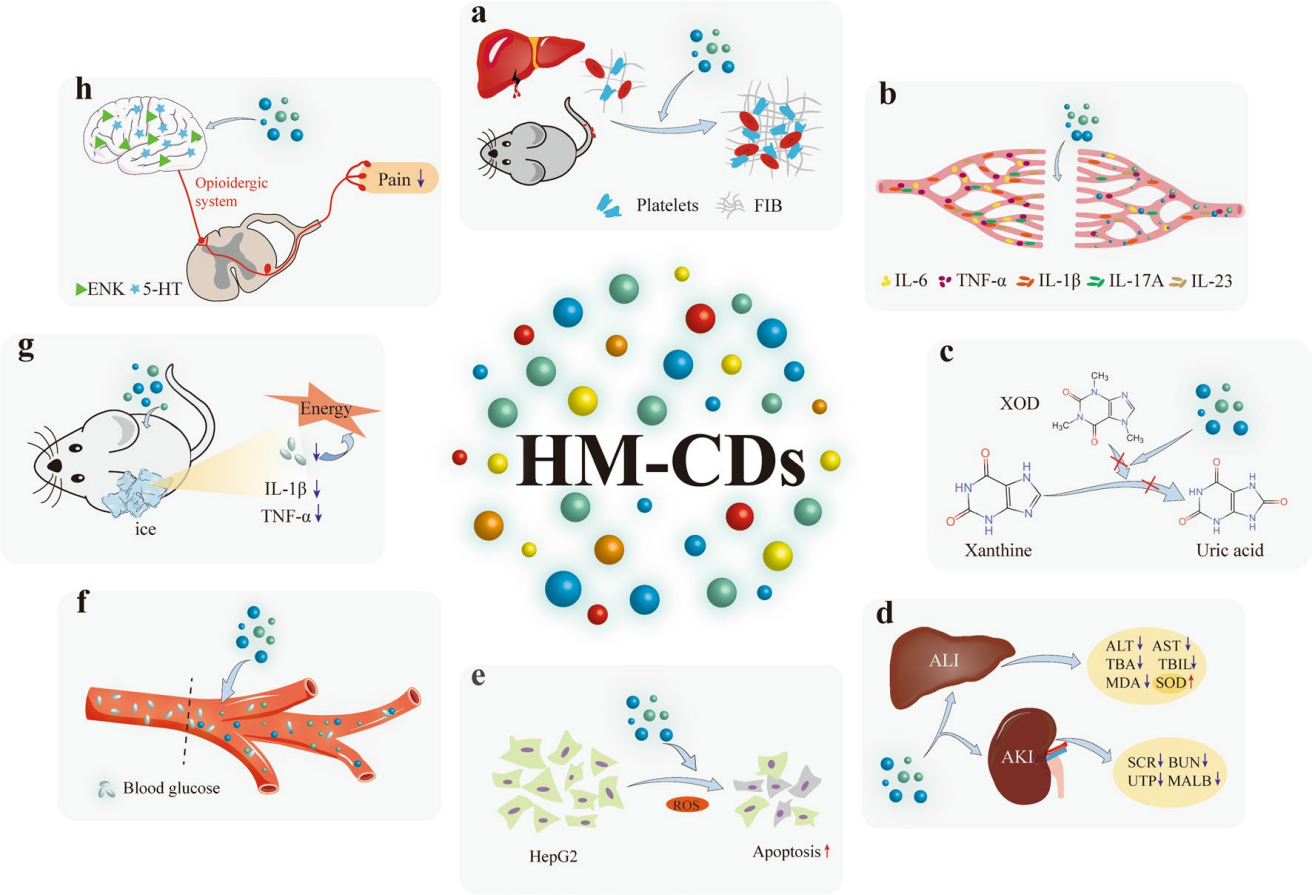
Medical applications and potential mechanisms of HM-CDs. **a** Hemostasis of HM-CDs, **b** Anti-inflammatory of HM-CDs, **c** Anti-hyperuricemia of HM-CDs. **d** Hepato-renal protective effect of HM-CDs, **e** Anticancer of HM-CDs, **f** Hypoglycemic of HM-CDs, **g** Anti-frostbite of HM-CDs, **h** Analgesic of HM-CDs. FIB: fibrinogen; XOD: xanthine oxidase; ALT: alanine transaminase; AST: acetone transaminase; TBA: total bile acid; TBIL: total bilium; MDA: malondialdehyde; SOD: superoxide dismutase; SCR: serum creatinine; BUN: blood urea nitrogen; UTP: urine total protein; MALB: microalbuminuria; ER: endoplasmic reticulum; ROS: reactive oxygen species; ENK: enkephalin; 5-HT: serotonin

Nanomedicine has become a highly active research field [105–107]. The emergence of CDs contributes new strength to the development of nanomedical science. Currently, avoiding complicated modifications and expensive materials receives key concerns. Existing CDs can be only treated diseases either by loading pharmacophores or as drug carriers. Excitedly, herbal medicine precursors may overcome these limitations through their specific efficacy, which naturally catches the eye of investigators. In this part, we discuss the diseases treated by existing HM-CDs and the specific functional mechanisms. (Fig. 10).

#### Hemostasis

Charcoal drugs, an essential component of herbal medicine, have more than 2000 years in China [108]. Although the hemostasis effects of charcoal drugs are broadly recognized, the material basis from the perspective of small molecule activators is poorly understood [108, 109]. The carbonization is completed by heating at high temperature, which is one of the operations of pyrolysis and microwave methods. HM-CDs can be prepared after the charcoal herbs are boiled, filtered and dialysis. In view of this, researchers turned their attention to the novel products, and simultaneously elucidated the specific mechanisms of HM-CDs in hemostasis.

*Junci Medulla Carbonisat*, *Pollen Typhae Carbonisata* and *Schizonepetae Herba Carbonisata* have long been incorporated into the hemorrhagic carbonized herbal medicine in the treatment of hemorrhagic diseases for hundreds of years. The experiments on tail haemorrhage and liver haemorrhage revealed that SHC-CDs and *Pollen Typhae Carbonisata*-CDs (PTC-CDs) attenuated activated partial thromboplastin time (APTT) and increased fibrinogen (FIB), indicating that they can exert hemostatic effects by activating the internal coagulation system and FIB [97, 109]. Another study showed that SHC-CDs may inhibit bleeding by increasing platelets [98]. In addition to activation of FIB system, the *Junci Medulla Carbonisata*-CDs (JMC-CDs) activate the exogenous coagulation system. It provides a perspective to explore the mechanisms of charred herbal medicine with hemostatic effects.

Apart from charcoal drugs, herbal medicine that dose not perform hemostatic effects can be prepared into CDs by pyrolysis, endowing them with additional hemostatic function. For example, egg yolk oil-CDs (EYO-CDs) suppressed bleeding by activating the intrinsic coagulation pathways and FIB system in a dose-dependent manner [44]. Another work [110] demonstrated that low doses of *Phellodendri Cortex Carbonisatus*-carbon dots (PCC-CDs) (1 mg/kg) significantly cut down thrombin time, and had an excellent hemostatic effect, thus reduced PCC usage in vivo.

#### Anti-inflammatory

**Fig. 11:**
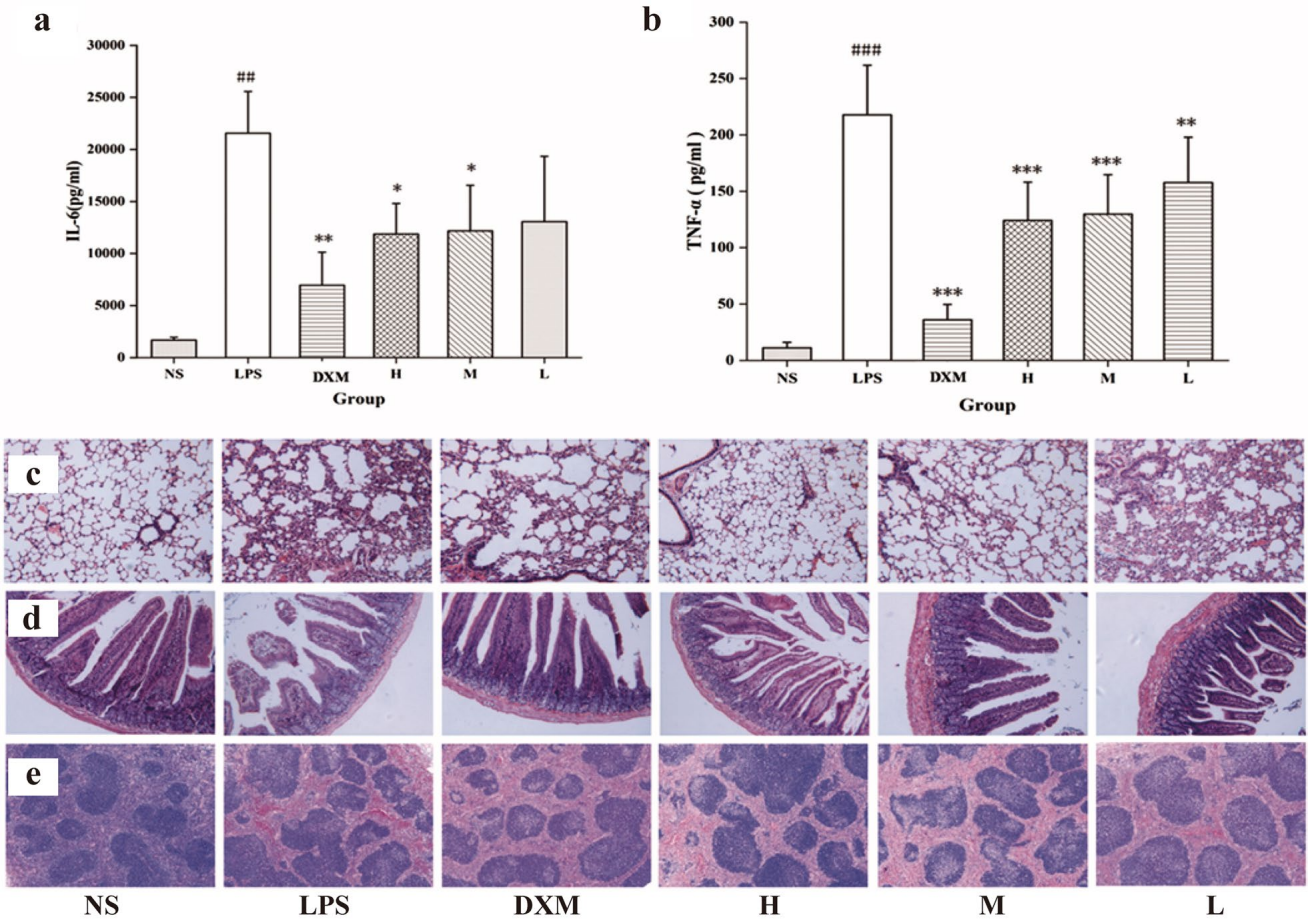
Effects of MSC-CDs on LPS-induced sepsis model. **a** Effects of MSC-CDs on IL-6. **b** Effects of MSC-CDs on TNF-α. **c** Effect on histopathological damage of lungs. **d** Effect on histopathological damage of small intestine. **e** Effect on histopathological damage of spleen. (n = 6, NS: normal control, LPS: model, DXM: 0.67 KU/kg, H: High-dose MSC-CDs (1.4 mg/kg), M: Middle-dose MSC-CDs (0.7 mg/kg), L: Low-dose MSC-CDs (0.35 mg/kg)). *p < 0.05; **p < 0.01, and ***p < .001 compared with model group; ##*p* < 0.01 and ###*p* < 0.001, compared with NS group. Reprinted with permission from ref. [45]. Copyright (2019) The Authors. Published by Informa UK Limited

Inflammation, a complex pathological process of multi-pathways and multi-molecules, corresponds to the pharmacological effects of multi-components and multi-targeting herbal medicines [111, 112]. Herbal medicine is known as a promising therapy to carry out anti-inflammation [113]. Despite increased anti-inflammatory bioactive substances from herbal medicine [114], the underlying mechanisms still needs to be lucubrated. To date, mulberry silkworm cocoon-CDs (MSC-CDs), *Lonicerae japonicae Flos*-derived CDs (LJFC-CDs), *Aurantii fructus immaturus carbonisata*-derived CDs (AFIC-CDs) and *Puerariae lobatae Radix* CDs (PLR-CDs) were produced through pyrolysis. Among these HM-CDs, MSC-CDs displayed favorable anti-inflammatory effects in the xylene-induced ear edema and acetic acid-induced vascular permeable mouse models, which provides clear evidence for inflammatory treatment related to vascular endothelial barrier leakage and cytokine release. More attractively, in the LPS-induced systemic inflammation model, MSC-CDs attenuated serum levels of IL-6 and TNF-α by dose-dependence [45] (Fig. 11).

Recently, Hu et al. [115] showed *Radix Sophorae Flavescentis* carbonisata (RSFC)-based CDs against an ethanol-induced acute gastric ulcer in rats by inhibiting TNF-α and IL-6 releases via downregulation of NF-κB pathway. RSFC has been extensively harvested for treating ulcerative diseases throughout the body. The authors concluded that HM-CDs using high-temperature pyrolysis may have inherent bioactivities [115]. But the active ingredients are not revealed.

With regard to anti-inflammation induced by LJFC-CDs, they markedly declined levels of IL-1β, IL-6 and TNF-α in LPS-induced heating models [116]. Besides, AFIC-CDs [117] and PLR-CDs [118] ameliorated the degree of joint swelling in gouty arthritis, and the former reduced the levels of IL-1β and TNF-α in a dose-dependent manner. Furthermore, treatment of psoriasis-like inflammation with PCC-CDs not only reduced the IL-6 and TNF-α, but also decreased the IL-17A and IL-23 levels. These findings offer a novel approach for anti-inflammatory of herbal medicine.

#### Anti-hyperuricemia

**Fig. 12:**
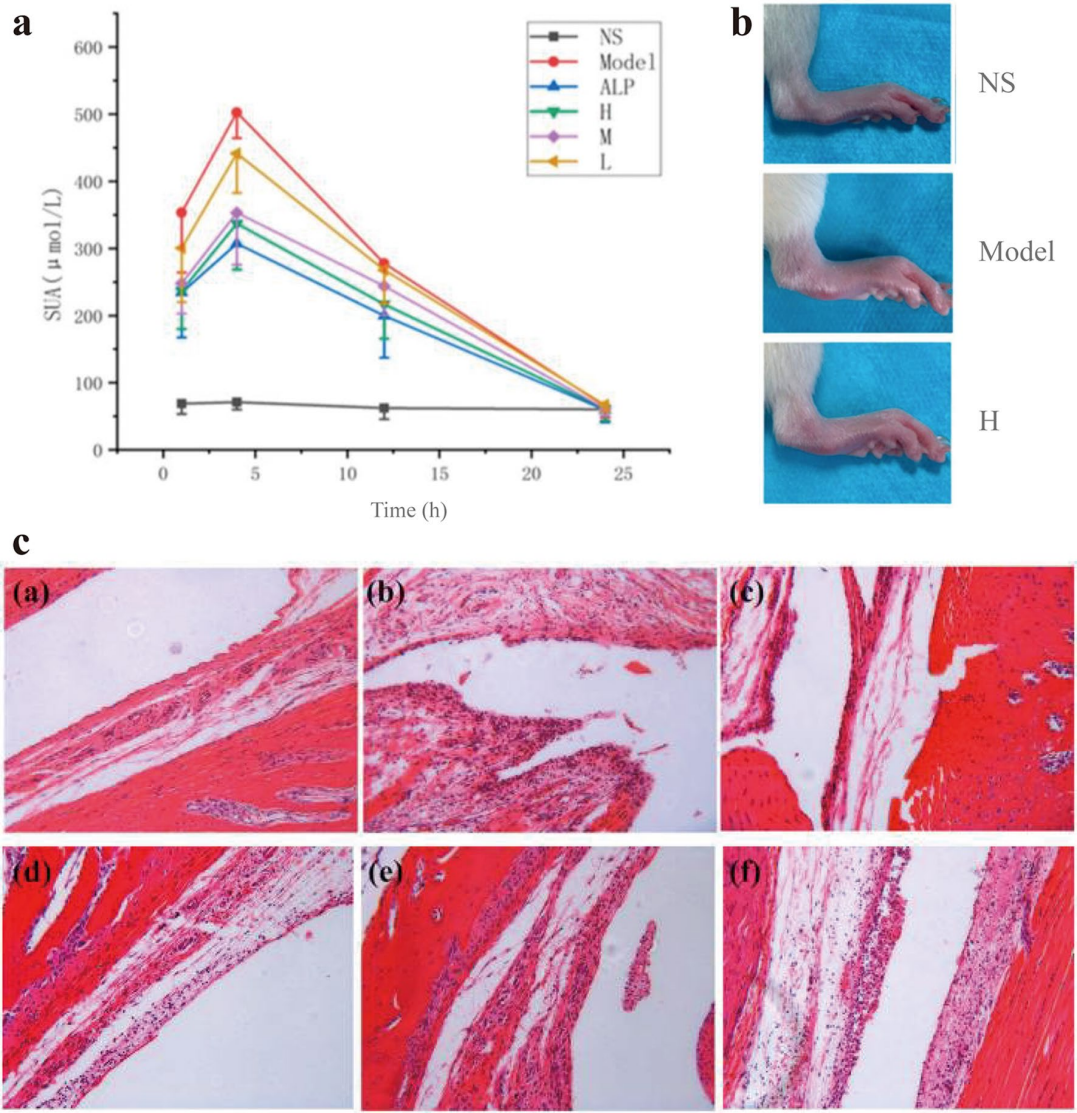
Anti-Gout effects of PLR-CDs. **a** Effects of PLR-CDs on serum uric acid levels for 24 h. **b** Representative images of joints from NS, Model, and H groups at 12 h. **c** Histological examination of H & E stained ankle joint tissues of normal group (**a**), model group (**b**), Col group (**c**), High-dose PLR-CDs group (**d**), Middle-dose PLR-CDs group (**e**), and Low-dose PLR-CDs group (**f**). Reprinted with permission from ref. [118]. Copyright (2019) by the authors. Licensee MDPI, Basel, Switzerland

Hyperuricemia is one of the pathological processes of gyration mainly associated with purine metabolic disorder and higher serum urate [119, 120]. The elevation of xanthine oxidase (XOD) is responsible for the pathological basis for the overproduction of uric acid in the kidneys. Allopurinol, an inhibitor of XOD, currently becomes a first-line drug for clinical treatment of gout and hyperuricemia [121]. Nevertheless, skin rash is the most common adverse effect of allopurinol, increasing high mortality [122, 123]. For the treatments, AFIC-CDs and PLR-CDs prepared by Zhao et al. [117, 118] reduced uric acid levels in a short period (Fig. 12), and also decreased inflammation during the acute phase of gout. Particularly, the non-toxicity of CDs effectively avoided the potentially toxic side effects of allopurinol [124]. Therefore, it offers a safer and more reliable regimen for gout and hyperuricemia in clinical practice.

#### Hepato-renal protective effect

In western countries, herbal medicine is often considered adequate only for chronic diseases and slow or ineffective for acute injuries [125]. However, HM-CDs confirmed the therapeutic effects of herbal medicine in acute injuries. In a latest study [126], the *Paeoniae Radix Alba* Carbonisata-derived CDs (PRAC-CDs) diminished alanine transaminase (ALT) and acetone transaminase (AST), and inhibited total bile acid (TBA) and total bilium (TBIL) in mice model of acute liver injury. The CDs led to a hepatoprotective effects via a decrease in Malondialdehyde (MDA) content and an increase in superoxide dismutase (SOD) by removing oxygen freelance, inhibiting lipid peroxidation of liver cells, and regulating the metabolism of bile acid. Another study revealed that PCC-CDs directly exerted renal-protection reversing increases of serum creatinine (SCR), blood urea nitrogen (BUN), total urine protein (UTP) and microalbuminuria (MALB) in acute kidney injury models [127]. The PCC-CDs also mitigated the inflammatory responses and thrombocytopenia associated with acute kidney injury and thereby acted multifacetedly.

#### Anticancer

Herbal medicine has been diffusely used in the alternative treatment of cancer, and further plays an auxiliary therapeutic role by regulating cancer genes and anti-cancer genes, epigenetic modifications, and tumor microenvironment [128–130]. HM-CDs execute anti-cancer effects which may be a new form for positioning tumors by photoluminescence. Following hydrothermal synthesis, Ginger-CDs excited strong blue, green and red fluorescence at different wavelengths [28]. Compared with CDs synthesized by EDTA, glycine and green tea, ginger-CDs selectively induced apoptosis by inducing ROS production in HepG2 cells [28]. This pharmacologic action was dependent on the surface modification of ginger-CDs. Simultaneously, ginger-CDs were excreted by urine within 1 h, and not be stored in large quantities in the body, resulting in avoidance of potential biotoxicity. Unexpectedly, the surface composition of ginger-CDs did not contain the 6-gingerol of the anti-cancer active component except curcumin [28]. On basis of this, some substances may be changed after high-temperature heating can be inferred, leading to diverse pharmacological effects between HM-CDs and herbal medicines. Hence, researchers need to continually explore and validate active pharmaceutical ingredients of HM-CDs. It must be pointed that few publications are reported on tumor bioimaging applications of HM-CDs. Part of the reason may lie in the deficiency of red HM-CDs required for bioimaging.

#### Hypoglycemic

*Jiaosanxian*, a charcoal herbal medicine, has the effect of eliminating food mass. The postprandial glycemia and insulin levels are inversely proportional to carbohydrate degradation rate [131]. So JSX-CDs were synthesized to lower blood glucose levels [132]. Fifteen minutes after gavage, JSX-CDs markedly reduced the blood glucose level of hyperglycemic mice. Although there was no significant difference between the JSX-CDs and control groups after 90 min, blood glucose level was still lower than those in the control group. Preliminary results revealed that JSX-CDs did not cause hypoglycemia in normal mice. But the underlying mechanism of action is still not precise.

#### Anti-frostbite

**Fig. 13:**
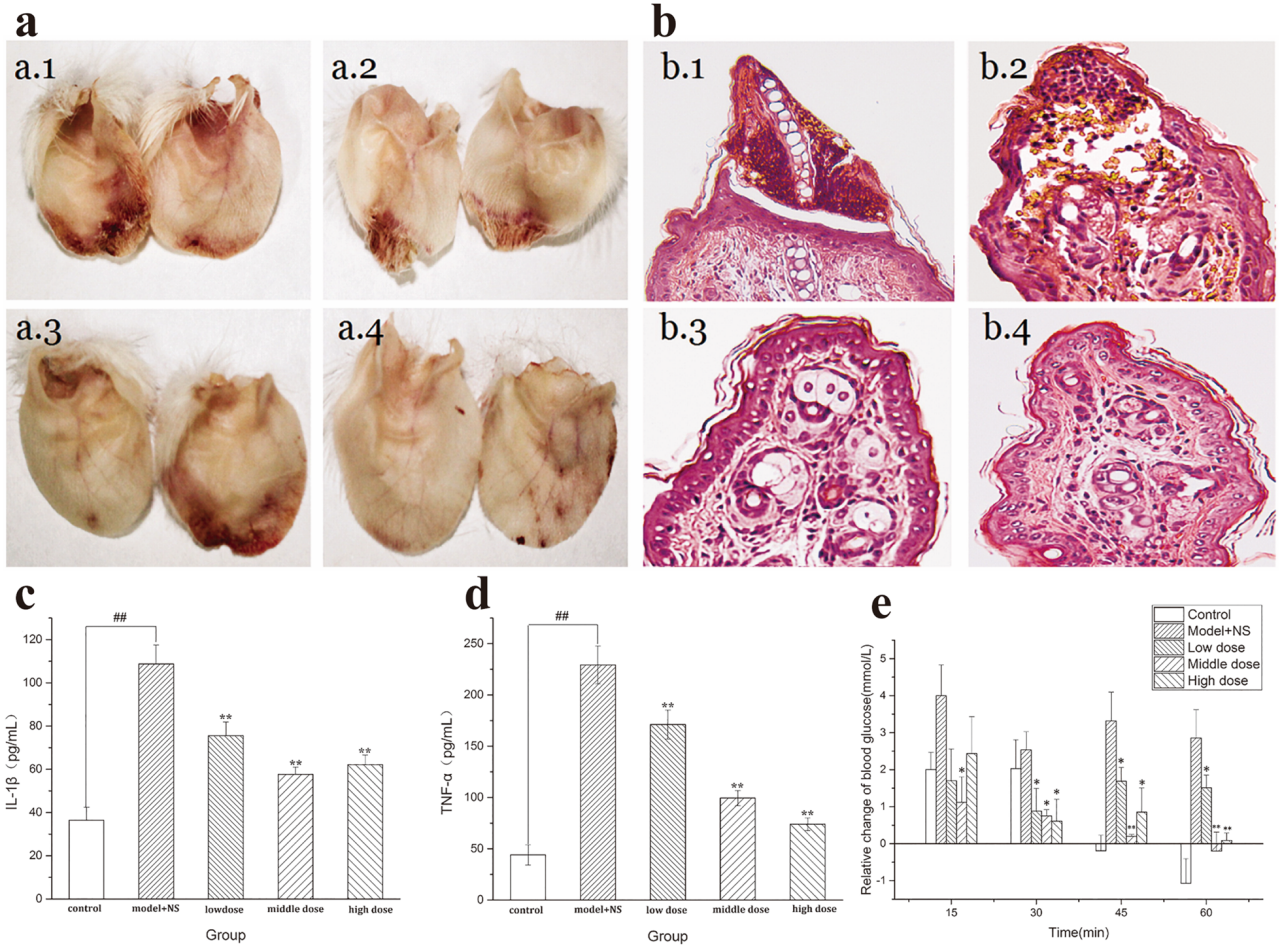
Effects of AAFC-CDs on frostbite in mice. **a** Morphology of mouse ear tips: a.1 Model + NS group; a.2 low-dose AAFC-CDs group; a.3 middle-dose AAFC-CDs group; a.4 high-dose AAFC-CDs group. **b** Histology of HE-stained mice ear tips: b.1 Model + NS group; b.2 low-dose AAFC-CDs group; b.3 middle-dose AAFC-CDs group; b.4 high-dose AAFC-CDs group. **c** Concentrations of IL-1β. **d** Concentrations of TNF-αin mouse sera. **e** Blood glucose levels. *p < 0.05 and **p < 0.01 compared with NS group. *p < 0.05 and **p < .01 compared with control group. Reprinted with permission from ref. [134]. Copyright (2020) The Authors. Published by Informa UK Limited

Frostbite caused by cold conditions triggers various degrees of damage to tissues, but interventions are lacking [133]. To bridge this gap, Kong et al. [134] synthesized *Artemisiae Argyi Folium* (AAF) Carbonisata-CDs (AAFC-CDs) by pyrolysis method. AAFC-CDs achieved anti-frostbite efficacy by mediating IL-1β and TNF-α to improve local inflammation and providing energy for the body to reduce blood glucose levels caused by frostbite (Fig. 13). Unlike traditional AAF, isochlorogenic acid no longer existed in AAFC-CDs, but the specific components have not been identified. Traditional AAF was not indicated for treating frostbite previously. Hence, the appearance of AAFC-CDs may expand the practical applications of AAF.

#### Analgesic

The analgesic activity of *Zingiberis Rhizoma* (*Ganjiang*, ZR), a processed product of ginger, has been for millennia. Zhang et al. [135] prepared the ZR-CDs by the high-temperature pyrolysis. ZR-CDs were comparable to the analgesic activity of morpholino [136]. The analgesic mechanisms on the following two aspects. First, ZR-CDs could enhance endogenous opioid peptide enkephalin (ENK), which was abrogated with non-selective antagonist naloxone, indicating that the activation of opioid system was one of the analgesic effects. Second, ZR-CDs increased serotonin (5-HT) in the brain tissues, but the content of 5-HT was decreased in the plasma, thus achieving dual regulation. The authors speculated that this might be associated with activating different 5-HT receptor subtypes, but explicit validation was not given. Crucially, no adverse effects were detected in vivo and in vitro. ZR-CDs supply a better green and safe analgesic strategy than opioids with side effects (respiratory suppression, drug dependence, etc.) [136].

HM-CDs have been investigated and documented from the above discussion to have considerable therapeutic effects in many diseases. Nevertheless, three questions remain. (i) Components of HM-CDs are a mystery. High-temperature conditions may cause the decomposition of herbal medicine, leading to reduction or even disappearance of active ingredients. The difference between HM-CDs and herbal medicine has not yet been clarified. (ii) Metabolic process in *vivo* is unknown. The essence of HM-CDs is nanoparticles, and the metabolic process is critical. Elucidating the metabolic mechanisms is one of the most notable challenges. (iii) Theranostic systems have not been realized. There is no report on HM-CDs as both an in vivo imaging agent and a therapeutic agent.

### Ion and molecule detection

**Fig. 14:**
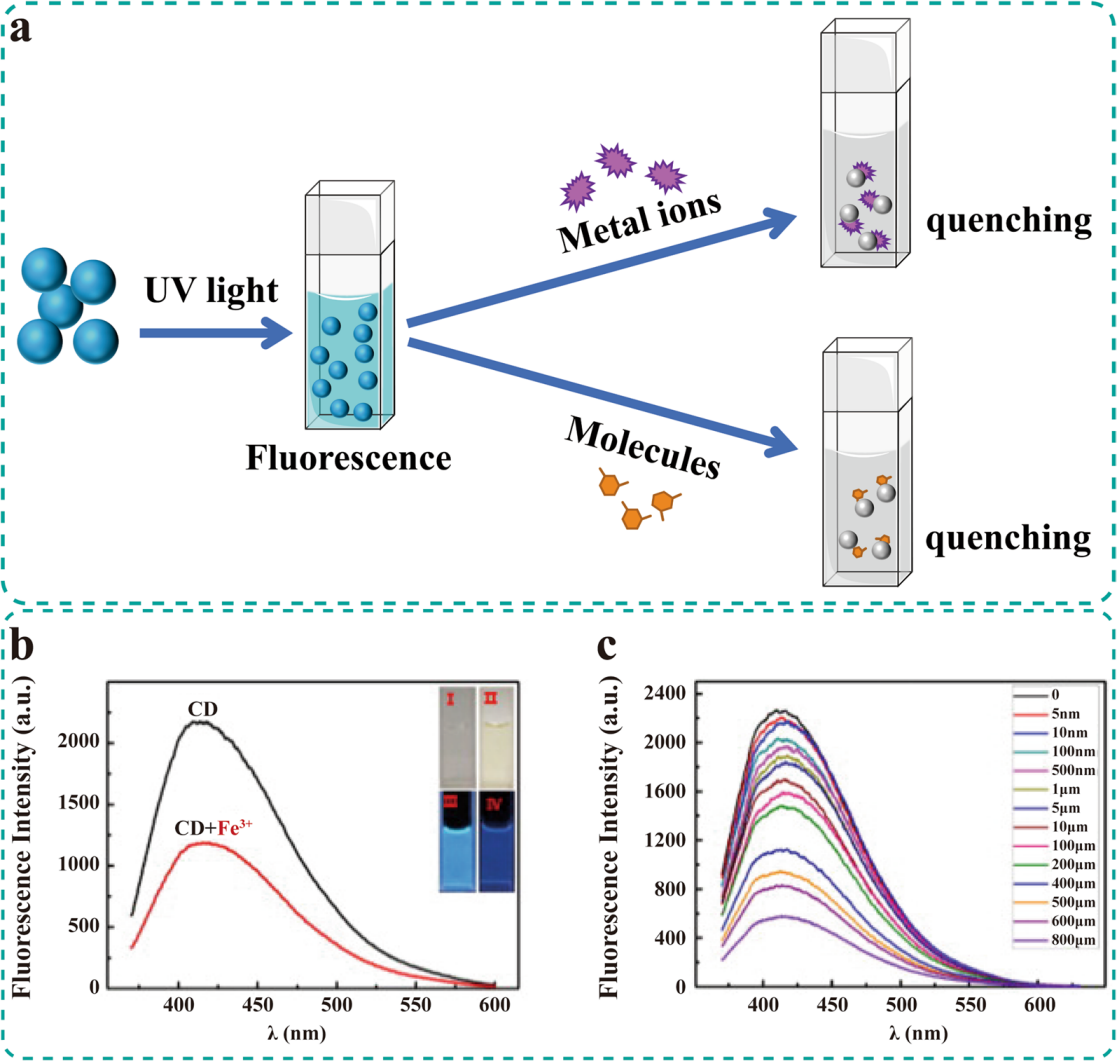
HM-CDs as sensing agents. **a** Binding of metal ions or molecules on CDs causes fluorescence quenching. **b** Fluorescence spectra of honey-CDs in the absence (black) and presence (red) of 100 μM Fe^3+^. Inset: photographs. **c** Fluorescence spectra of honey-CDs in the presence of different concentrations of Fe^3+^. Reprinted with permission from ref. [43]. Copyright (2014) Elsevier

Fe^3+^, Cu^2+^, Al^3+^ and Ag^+^ are abundant transition metal ions in biological systems. These ions play vital roles in many physiological and pathological processes, such as cell metabolism, cell proliferation, catalysis and DNA synthesis [137]. Some ions have been confirmed to fluctuate with the occurrence of a specific disease. For instance, aberrant fluctuation of Fe^3+^ is a marker of chronic heart failure [138]. However, excessive amounts of metal ions from ingestion of contaminated water and food can be highly toxic to organisms. Therefore, it is necessary to test the level of metal ions, such as Cr^6+^ and other toxic metal ions, in living organisms and everyday products. Nowadays, methods such as atomic absorption spectrometry [139], inductively coupled plasma mass spectrometry [140] and electrochemistry [141] have been utilized to detect metal ions. But these techniques are expensive and complex [80, 142]. A safe, efficient, sensitive and reliable means need to be developed. Owing to the high fluorescence stability, CDs play a unique strength in molecular and ion detection. When CDs combine with molecules or ions, their energy will be transferred, affecting the fluorescence properties (Table 3). Typically, the fluorescence quenching depends on several mechanisms, including the inner filter effect (IFE), fluorescence resonance energy transfer (FRET), static quenching effect, dynamic quenching effect and electron transfer [143–147]. This section discusses the application and sensing mechanisms of HM-CDs in ion and molecular detection (Fig. 14a).

**Table 3:** HM-CDs as sensors

Herbal medicine	Iron/molecule	Sensing mechanisms	Refs
Jujubes	Fe^3+^	Electron transfer	[149]
Honey	Fe^3+^	Electron transfer	[43]
Lycii Fructus	Fe^3+^	IFE/electron transfer/energy transfer	[148]
Pinellia Ternata	Cr^6+^	Synergistic effect of IFE and electron transfer	[151]
*Osmanthus fragrans*	Al^3+^/quercetin	IFE and static quenching effect	[152]
*Osmanthus fragrans*	Fe^3+^	IFE	[150]
Mustard seeds	Ascorbic acid	Electron transfer	[157]
Ginkgo leaf	Salazosulfapyridine	IFE	[85]
Papaya	Fe^3+^/*Escherichia coli*	Electron transfer/FimH proteins interact with mannose	[78]
*Purple perilla*	Ag^+^	Electron transfer	[142]
Orange peel	Cr^6+^	IFE	[84]
Orange peel	*Escherichia coli*	Aptamer to *E. coli* cells	[103]
Lychee seeds	Methylene blue	Electron transfer	[96]
Rose flower	Tetracycline	Energy transfer	[153]
Giant Knotweed Rhizome	Hg^2+^	Energy transfer	[87]
Bamboo leaves	Cu^2+^	IFE	[80]
*Panax notoginseng*	Cr^6+^	IFE	[38]
Hair	Fe^3+^/ATP/NAPDH	Synergistic action of the metabolism process by the digestive system and quenching effect of internal metabolite and substance	[42]
Yams	Hg^2+^/6-mercaptopurine	FRET/decreased conjugation	[79]
Gardenia fruit	Hg^2+^/cysteine	Static quenching	[88]
Coix seed	Furazolidone	Static quenching/IFE	[82]
Smilax China	Cu^2+^	Static quenching	[147]

IFE: inner filter effect; FRET: fluorescence resonance energy transfer

#### Detection of Fe^3+^

Wang et al. [78] prepared two kinds of papaya-CDs while water and ethanol were used as the solvent (W-CDs and E-CDs) for selective Fe^3+^ detection. In different concentrations of Fe^3+^, the fluorescence emission of CDs at 450 nm decreased, which was since both CDs contain sufficient oxygen-containing functional groups (-OH and -COOH) to form complexes with Fe^3+^. Moreover, the content of oxygen-containing functional groups in E-CD was higher than that in W-CD, so E-CD was more sensitive (detection limit, DL, was 0.29 μM). The detection performance was confirmed to be comparable with the Phenanthroline spectrophotometry by detecting iron in the heme capsules. Another CD consistent with this fluorescence quenching is honey-CDs. Yang et al. [43] applied honey-CDs to detect Fe^3+^ based on the direct interactions between Fe^3+^ and –COOH, –OH and –NH_2_ (Fig. 14b, c). The DL of Fe^3+^ was 1.7 × 10^–9^ mol/L at a signal-to-noise ratio of three. The standard recovery experiments noted that the recoveries of human blood samples were 96.00%, 97.25% and 101.50%, which demonstrated the utility of honey-CDs [43].

Sun et al. [148] synthesized the water-soluble CDs with *Lycii Fructus* (LF-CDs). LF-CDs can selectively and sensitively detect Fe^3+^. The DL was estimated to be 21 nM. The absorbance intensity increased with Fe^3+^ concentrations, indicating that IFE might be one reason for fluorescence quenching. Additionally, LF-CDs chelated Fe^3+^ with abundant surface functional groups, such as C–OH and C–O–C. The complexes accelerated the nonradiative electron/hole recombination through effective photoelectron or energy transfer, resulting in further fluorescence quenching [148]. The fluorescence quenching of hair-derived CDs was consistent with LF-CDs. Fe^3+^ was also detected using other HM-CDs, such as CDs derived from *jujubes* via electron transfer [149], *Osmanthus fragrans Lour*-CDs via IFE [150].

#### Detection of Cr^6+^

Dai et al. [151] used *Pinellia ternata* as a precursor and ethylenediamine as a nitrogen source to synthesize nitrogen-doped *Pinellia* quantum dots (PTN-CQDs). This CD detected Cr^6+^ selectively and sensitively in different water samples, and the DL was 15 nM. In addition to IFE, Cr^6+^ was easy to capture electrons because of the strong electron acceptance and affinity. As a result, when the excited state molecules of PTN-CQDs collided with Cr^6+^, it was highly prone to complexes, causing electron transfer and nonradiative transitions, leading to fluorescence quenching.

To ameliorate the selectivity of CDs, Wang et al. [84] used EDTA (a metal chelator) to modify an orange peel quantum dot (CDs@EDTA). They only found that IFE but not electron transfer was associated with fluorescence quenching. Furthermore, *Panax notoginseng* carbon dots (Pn-CDs) also detected Cr^6+^ via IFE, and the DL was 0.185 nM [38].

#### Detection of Hg^2+^

Li et al. [79] used Chinese yams as a source of carbon and nitrogen to prepare water-soluble yam quantum dots and modified the surface with carboxyfluorescein (FAM)-DNA macro-molecules (NCDs-ssDNA) to promote strong π–π accumulation. When 6-mercaptopurine (6-MP) was added, it could form a large conjugated rigid plane structure with FAM-DNA, resulting in fluorescence enhancement. However, the addition of Hg^2+^ terminated this reaction because the binding of Hg^2+^ to DNA disrupted the original system. This response pattern is called “on–off-on sensing mode.”

Similarly, Sun et al. [88] produced the nitrogen and sulfur co-doped carbon dots (N/S-CDs) using gardenia fruit without any surface modifications. N/S-CDs were used to detect Hg^2+^ and cysteine. Hg^2+^ and S^−^ could form Hg^2+^-S bonds, and caused fluorescence to decrease. Subsequently, due to the strong interaction between Hg^2+^ and the sulfhydryl group of cysteine, the Hg^2+^-S bond was destroyed after adding cysteine to the above system, thereby restoring fluorescence.

#### Detection of Al^3+^

Yu et al. [152] used *Osmanthus fragrans* to synthesize nitrogen-doped quantum dots to detect quercetin and Al^3+^. When Al^3+^ was added to the CDs and quercetin system, it was easier to form complexes due to the strong binding force between Al^3+^ and quercetin, which naturally eliminated the IFE effect and restored fluorescence [152]. Meanwhile, the human bladder cancer T24 cells viability remained more than 90% when incubated at 1000 μg/mL CDs concentration. The CDs undoubtedly provide a green and safe means to detect Al^3+^ and quercetin in the biological field.

#### Detection of Cu^2+^

Liu et al. [80] synthesized CDs using bamboo leaves and modified them with branched polyethyleneimine (BPEI) with a robust chelating effect (BPEI-CDs). The BPEI-CDs were used for detecting Cu^2+^ in environmental water. The DL was as low as 115 nM, and the dynamic range was 0.333–66.6 μM. The quenching principle was attributed to IFE of the copper amine complex formed at the surface of BPEI-CDs [80]. It offered a green, safe and high-efficient alternative approach for detecting Cu^2+^ in the water, especially industrial wastewater. Another work synthesized Smilax China-derived yellow-fluorescent CDs (y-CDs) by hydrothermal method [147]. Based on the static quenching effect, Cu^2+^ was highly selective detected (among 23 ions and 14 amino acids) with linear ranges of 0.5–10 μM, 75–225 μM and 250–350 μM, achieving a DL of 28 nM [147].

#### Detection of Ag^+^

Zhao et al. [142] prepared *purple perilla* CDs with low toxicity and biocompatibility by hydrothermal synthesis. The CDs detected Ag^+^ sensitively and selectively with a DL of 1.4 nM. Nonradiative electron transfer that occurs from the excited state to the d orbital of Ag^+^ caused the fluorescence quenching. To obtain better sensing performances, this work optimized the experimental conditions, as follows. The pH was adjusted at 7.0, reaction time was 15 min, the temperature was maintained at 30 °C, and the concentration of CDs was 300 μg/ml. Choosing CDs as fluorescence sensors requires constant exploration of optimal reaction conditions to ensure stable preparation in the future.

#### Detection of molecules

Due to the green and safety of herbal medicine, it is well suited to detect certain toxic substances in food, drinking water and other resources.

*Detection of methylene blue* Xue et al. [96] used CDs derived from lychee seeds (LS-CDs) to detect methylene blue (MB) which could be adsorbed on the surface of LS-CDs, causing fluorescence quenching. LS-CDs are an encouraging means to detect toxic MB in wastewater because of low toxicity.

*Detection of salazosulfapyridine* Jiang et al. [85] produced nitrogen-doped quantum dots derived from ginkgo leaves to detect salazosulfapyridine (SASP). SASP could absorb the excitation of CDs to cause fluorescence quenching via IFE. Furthermore, the fluorescence lifetime of CDs was not related to SASP, which means that static quenching was also the possibility.

*Detection of tetracycline* CDs derived from rose flowers were adopted for detecting tetracycline via interaction [153]. This interaction resulted from the captured energy from CDs by forming new bonds with tetracycline, thus quenching fluorescence effectively.

*Detection of furazolidone* Furazolidone is a nitrofuran antibiotic but causes side reactions once excessive use. A highly fluorescent coix seeds CD was prepared for detecting furazolidone with a linear range of 0.5–100 μM [82]. The static quenching and IFE were possible quenching mechanisms [82]. As a novel fluorescence probe, coix seed-CD has simplicity, high selectivity and sensitivity relative to existing approaches (e.g., high-performance liquid chromatography, liquid chromatograph mass spectrometer, liquid chromatography with tandem mass spectrometry, electrochemical methods).

Although HM-CDs have been available for ion and molecule detection in some biological samples, other ions and molecules remain constantly explored. The water source is currently the primary sample tested by HM-CDs. Expanding the detection of substances in biological tissues using HM-CDs is highly warranted, such as blood, urine, saliva, tears and other liquid tissues convenient for collection. Other than that, it is also necessary to develop a sensing system for solid tissues. For example, detecting specific ions and molecules in brain tissue can rely on the BBB permeability of HM-CDs and the fluorescence quenching to achieve a safe and effective diagnosis and even treatment.

### Bioimaging

HM-CDs have better biocompatibility, availability and sustainability than chemical-CDs [23, 154], further extending the scope of biomedicine. In this section, we summarize the applications of HM-CDs in bioimaging (Table 4).

**Table 4:** HM-CDs in bioimaging

Herbal medicine	Organisms	Fluorescence color	Refs
Honey	HepG2/HeLa cells	Green	[43]
Lycii Fructus	HeLa cell	Blue/Glaucous/Green	[148]
Lemon juice	Onion epidermal cell	Blue-green	[155]
Ginkgo fruits	HeLa/KYSE410 cells	Blue/Green	[81]
Lychee seeds	HepG2 cell	Blue	[96]
Watermelon peel	HeLa cell	Green	[33]
Ginger	MCF-10A/A549/MDA-MB-231/HeLa/HepG2 cells	Blue/Green/Red	[28]
*Panax notoginseng*	*E. coli, B. cereus, S. cerevisiae*/onion epidermal cells/leaf of *N. physaloides*/BALB/c mice	Blue/Green/Red	[38]
*Osmanthus fragrans*	T24 cell	Blue	[152]
*Osmanthus fragrans*	A549 cell	Blue	[150]
Mulberry silkworm cocoon	Zebrafish/embryos	Blue/Green/Red	[45]
Hair	Zebrafish/embryos	Blue/Green/Red	[42]
Coix seed	Furazolidone	Blue/Green	[82]
Smilax China	PC12 cell	Blue/Yellow/Red	[147]
Papaya	HeLa cell	Blue/Green	[78]
*Purple perilla*	HeLa cell	Blue	[142]

#### Cellular imaging

**Fig. 15:**
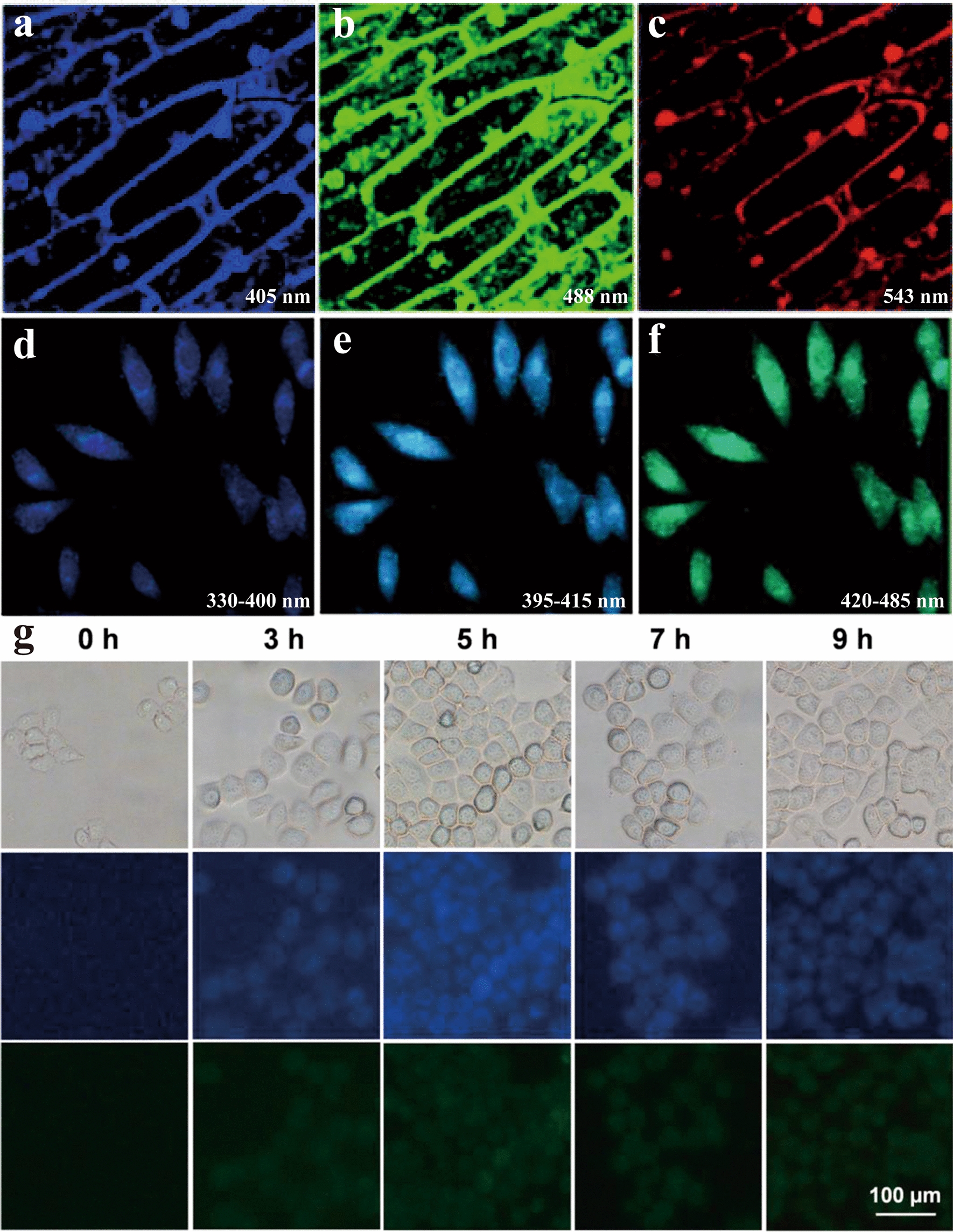
Cellular imaging applications of HM-CDs. **a**–**c** CLSM image of onion skin cells with Pn N-CDs at a 405 nm laser, 488 nm laser, and 543 nm laser. Reprinted with permission from ref. [38]. Copyright (2020) Royal Society of Chemistry. **d**–**f** Fluorescence microscope images of LF-CDs under UV-filter (330–400 nm), V-filter (395–415 nm) and B-filter (420–485 nm). Reprinted with permission from ref. [148]. Copyright (2017) Elsevier. **g** Fluorescence images of HeLa cells incubating with 1 mg/mL of W-CDs for 0 h, 3 h, 5 h, 7 h and 9 h. 1st row: bright field images; 2nd row: the images with an excitation/emission wavelength at 340 nm/420 nm; 3rd row: the images with an excitation/emission wavelength at 495 nm/520 nm. Reprinted with permission from ref. [78]. Copyright (2016) Elsevier

Due to the nanoscale size, HM-CDs are highly prone to be absorbed by cells, making them suitable for fluorescence imaging (Fig. 15) [154]. After labeling HeLa cells with LF-CDs, bright blue, glaucous and green luminescence were observed under the ultraviolet filter, V filter and B filter, respectively [148]. With the subsequent addition of Fe^3+^, the fluorescence intensity was decreased significantly. The lemon juice CDs were pursued as an imaging agent of onion epidermal cells [155]. The three-dimensional structures of the cell wall and cell nucleus were visible, and no adverse effects on the organisms. After incubating HeLa and KYSE410 cells with H-N-CDs for 8 h, blue and green fluorescence were observed under excitation at 405 nm and 488 nm, separately [81]. By comparing the fluorescence intensity of the nucleus and cytoplasm, researchers revealed that the CDs mainly entered into the cytoplasm. Although H-N-CDs did not cause cytotoxicity, whether fluorescence can discriminate different cells has not been confirmed.

In addition, Xue et al. [96] used LS-CDs to intervene in HepG2 cells. LS-CDs could penetrate cells and emit blue fluorescence to label cell membranes and cytoplasm. Other CDs used for imaging of HepG2 or HeLa cells also contained honey-CDs [43] and watermelon peel-CDs [33]. Of course, HM-CDs have been more versatile for other cell lineages imaging (Table 4). All above HM-CDs maintained the high viability of normal cells. However, researchers did not give us an indication of targeting of HM-CDs.

#### Other imaging

**Fig. 16:**
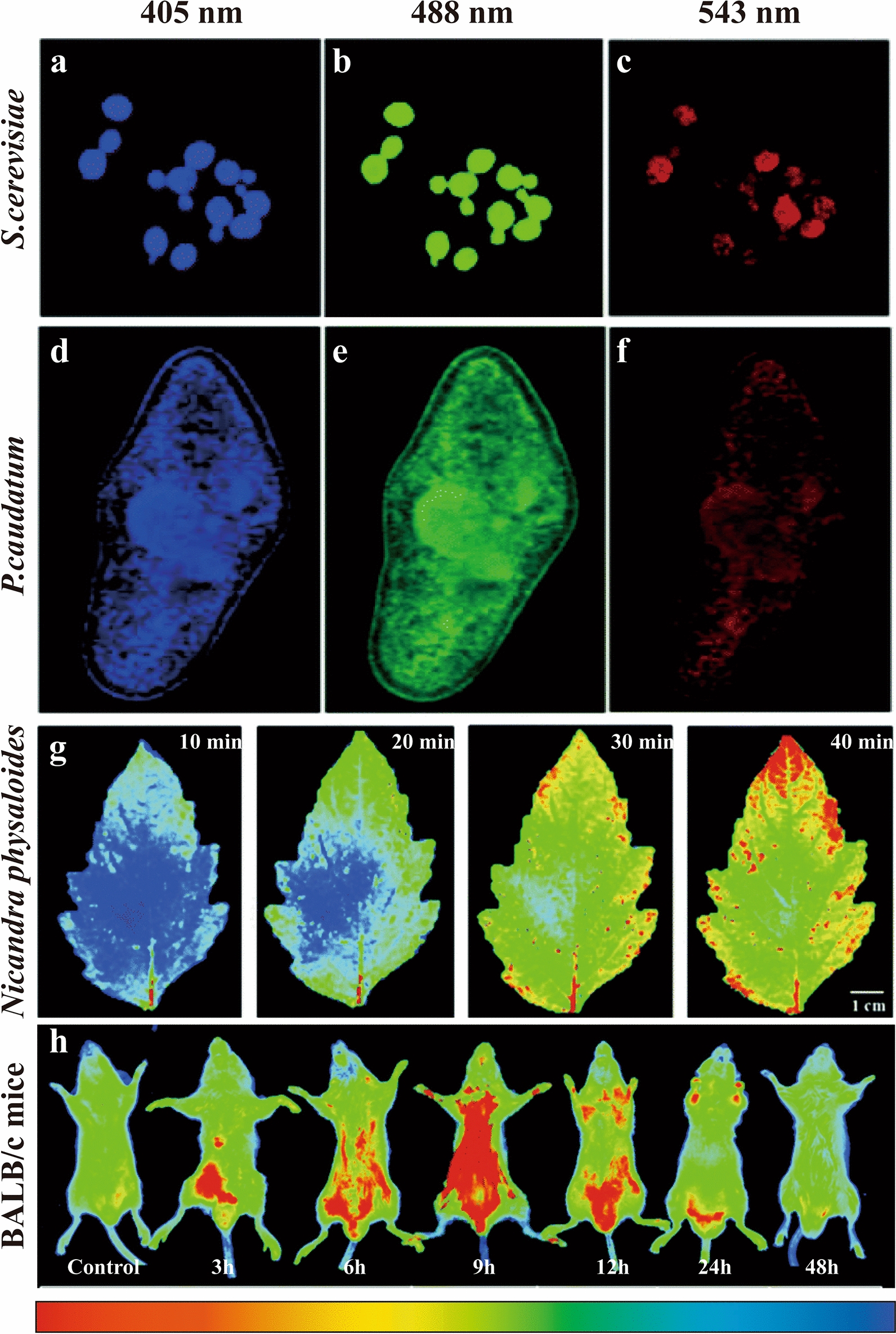
Other imaging applications of HM-CDs. **a**–**c** CLSM images of *S. cerevisiae* incubated with Pn N-CDs at 405 nm, 488 nm, 543 nm laser, **d**–**f** CLSM images of *P. caudatum* incubated with Pn N-CDs at 405 nm, 488 nm, 543 nm laser, **g** Imaging of *N. physaloides* with the injection of Pn N-CDs at different time intervals, **h** In vivo imaging of male BALB/c mice after injection of Pn N-CDs at different time intervals. Reprinted with permission from ref. [38]. Copyright (2020) Royal Society of Chemistry

Next to cellular imaging, few HM-CDs were applied to other biological imaging. A classic example is nitrogen-doped Pn-CDs [38], which was used for fluorescence imaging of multiple organisms. In microorganism imaging, Pn-CDs clearly labeled *E. coli, B. cereus and S. cerevisiae*. The blue, green and red fluorescence were observed at excitation wavelengths of 405, 488 and 543 nm, respectively (Fig. 16a–c). As the size of the microorganism increased, the fluorescence intensity was also enhanced. Pn-CDs marked the cell membrane and nuclear membrane in protozoan imaging by explicitly identifying the cell membrane structure or phospholipid bilayer of *P. caudatum* (Fig. 16d–f). In plant imaging, Pn-CDs spread along the veins of Arabidopsis thaliana under the induction of transpiration. Finally, they realized the distribution of whole leaves by spreading in the intercellular space (Fig. 16g). In animal imaging, the fluorescence intensity of Pn-CDs in BALB/c mice first increased and then decreased with increasing time. Pn-CDs were distributed throughout the body through the bloodstream, gradually absorbed by the stomach, and finally excreted through the intestine and bladder (Fig. 16h). Most notably, there was fluorescence expression in the brain at 9 h, which means that Pn-CDs were able to penetrate the BBB. This performance is significantly better than macromolecules. Thus, HM-CDs may offer a novel mode of administration for herbal medicine to treat brain diseases.

Although HM-CDs are poorly studied in bioimaging, they will provide a safe and reliable novel strategy in the future. Exploiting the potential of HM-CDs in live imaging is necessary, mainly in disease-location, which will contribute to the successful construction of theranostics of HM-CDs.

### pH sensing

**Fig. 17:**
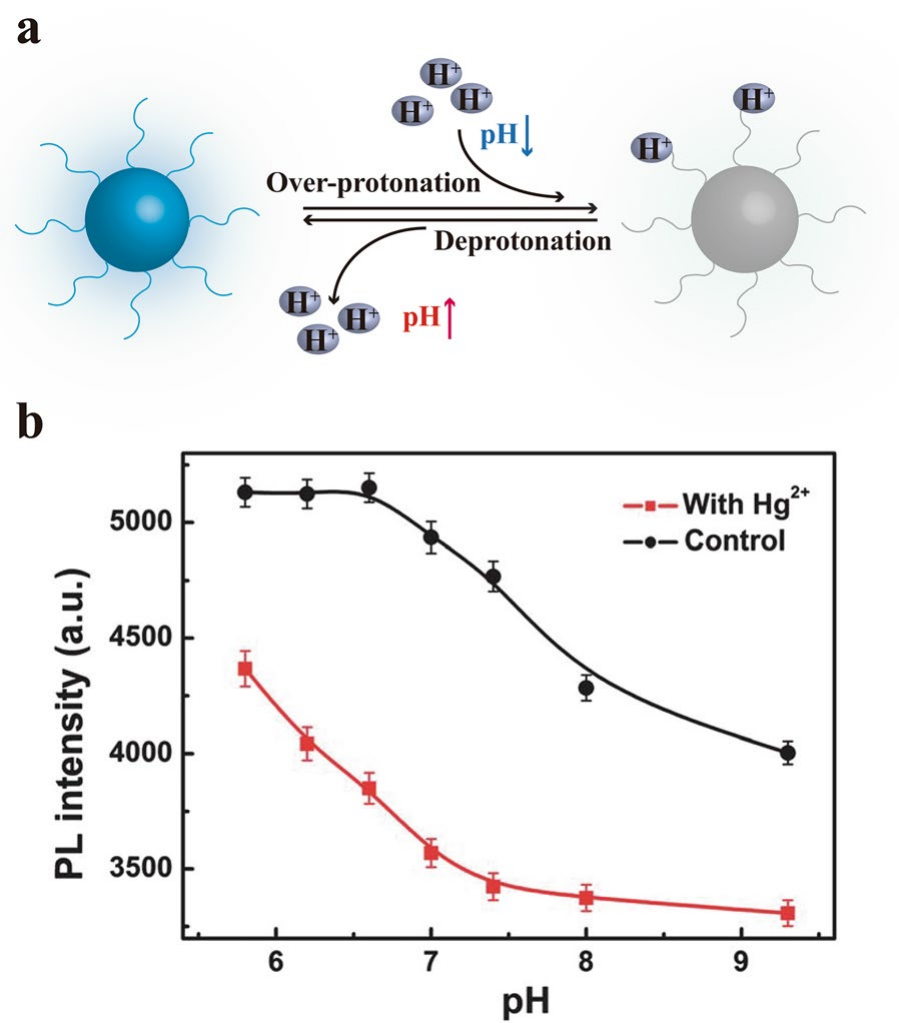
pH sensing of HM-CDs. **a** The response mechanism of pH-sensitive HM-CDs, **b** pH dependence for Hg^2+^ detection using photoluminescent CDs derived from *giant knotweed rhizome*. c(Hg^2+^): 100.0 mM; pH: 5.8, 6.2, 6.6, 7.0, 7.4, 8.0, 9.3; ex: 320 nm; em: 400 nm. Each point is an average of three successive measurements. Reprinted with permission from ref. [87]. Copyright (2013) Royal Society of Chemistry

Different diseases or body parts have various pH values [156]. Some of the HM-CDs have shown superior pH-responsive properties (Fig. 17), making them more suitable for different pH conditions. BPEI-CDs derived from bamboo leaves demonstrated a pH-dependent increase in fluorescence intensity in the pH range of 2–4 and decreased as pH further increased [80]. In a highly acidic (pH < 4) solution, the over-protonation of PEI led to diminished fluorescence, but the opposite was true at pH > 4 due to the deprotonation of PEI. This phenomenon was caused by the formation of intramolecular or intermolecular hydrogen bonds by functional groups on the surface of HM-CDs, such as N–H and O–H. Besides, CDs derived from fennel seeds [94], *giant knotweed rhizome* [87], *pinellia ternata* [151], mustard seeds [157], papaya [78] and *Purple perilla* [142] were also sensitive primarily to pH. Within these HM-CDs, *giant knotweed rhizome*-CDs efficiently detected Hg^2+^ based on photoluminescent from pH 5.8 to 9.3 (Fig. 17) [87] owing to the hydroxyl and carboxylic groups. It signifies that *giant knotweed rhizome*-CDs warrant as an Hg^2+^ fluorescent nanoprobe in either an acidic or alkaline medium.

## Conclusions and outlook

This review firstly systematically summarizes the research advances on HM-CDs by focusing on their synthetic approaches and specific applications. As a new branch of CDs, HM-CDs have been used in disease treatment, ion and molecular detection, bioimaging and pH sensing. The potential therapeutic effect is a meaningful sign that differentiates HM-CDs from other CDs. The medicinal value of herbal medicine may cause HM-CDs to contain medicinal substances without loading drugs, that may effectively avoid harmful effects. Furthermore, the application of HM-CDs in bioimaging has laid a solid foundation for theranostics. We expect that HM-CDs will make significant progress in the near future. Besides, the following questions also need to be addressed:

*More efficient and stable synthesis* Currently, two most common approaches for synthesizing HM-CDs are hydrothermal synthesis and high-temperature pyrolysis. Even though this “one-key” synthesis broadens the scope of HM-CDs’ applications, the QY, particle size, and fluorescence intensity are not stable. A more convenient and efficient microwave method is gradually appreciated which not only guarantees ecology and safety, but also enhances the efficiency of synthesis. More efficient and stable approaches require further development for efficient synthesis in the future.

*Identification of active ingredients* Although HM-CDs have unique advantages for therapy, their intrinsic effective ingredients are still uncertain. Active ingredients are essential to treat diseases. Identification of the effective substances is the direct evidence to elucidate mechanisms of action of HM-CDs. Existing synthesis methods may cause the decomposition or even induce disappearance of some practical components of herbal medicine at high temperatures. Therefore, remaining moieties of active compounds on HM-CDs under different synthesis methods and conditions is an important research direction in the future. Meanwhile, establishing an identification way to explore pharmacodynamic constituents of HM-CDs is the key to the subsequent research. At present, an effective strategy may be the high-performance liquid chromatography tandem mass spectrometry (HPLC–MS), which can be used as an available means for identifying pharmacodynamic constituents of HM-CDs.

*Clarifications of metabolism and distribution in vivo* The metabolism and distribution of nanomaterials have always been a hot issue. Although HM-CDs have shown excellent safety at the cellular level, studies on metabolism and distribution are still scarce. In vitro toxicity tests are far from sufficient to prove the safety of HM-CDs throughout the biological system. Future studies should manage to answer the following four questions: (i) What are HM-CDs' metabolic pathways in vivo? (ii) How are HM-CDs distributed in vivo? (iii) Will different herbal precursors be accompanied by different metabolism and distribution in vivo? (iiii) After receiving long-term treatment, whether HM-CDs deposit in vivo and cause chronic toxicity? More complete in vivo toxicity tests, distribution and metabolism in vivo need to preferably reveal the unique functions of HM-CDs in organisms.

**Fig. 18:**
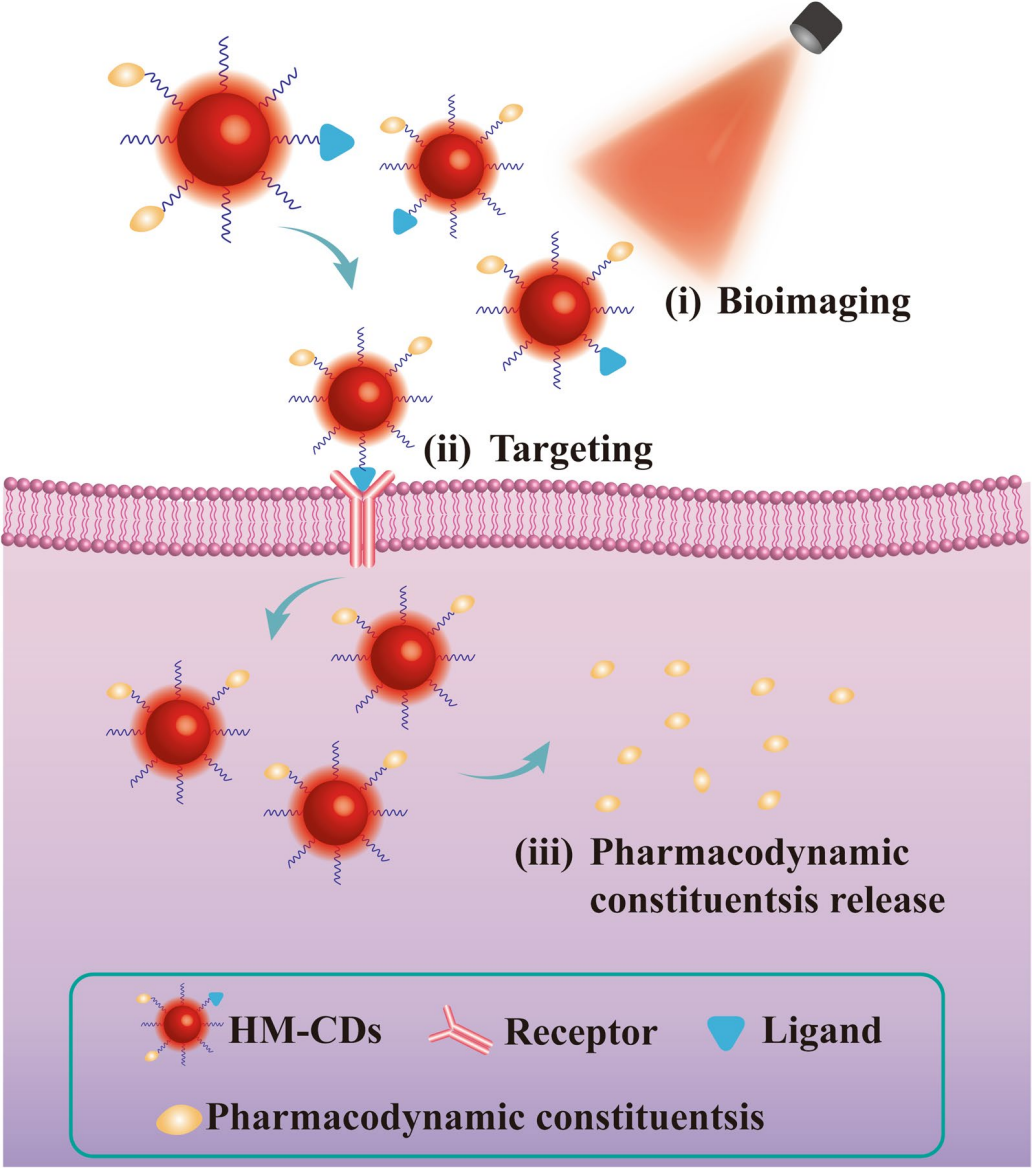
The future theranostics strategies in the field of HM-CDs

*Realization of theranostics* The photoluminescence of CDs is one of the most convincing characteristics. Research on the integration of diagnosis and treatment of HM-CDs seems boundless owing to the therapeutic effects. According to existing studies, the therapeutic effects and bioimaging are separate from each other. Moreover, the investigation of bioimaging remains at a very preliminary stage. The combination of therapy and bioimaging will provide unlimited possibilities for realizing theranostics which becomes a huge challenge: (i) The premise of safety and simplicity are the guarantees of optimal targeted therapy of HM-CDs. (ii) The red-emission CDs exhibit little damage to the biological matrix, deep tissue penetration and minimum autofluorescence background of biosamples [158]. Therefore, synthesizing red HM-CDs is required for bioimaging applications stably and efficiently. (Fig. 18) (iii) Aberrant mitochondrial function is involved in a range of human diseases [159]. Thus, the imaging in mitochondria of HM-CDs is also the sharp focus in the future.

*Exploration of different kinds of herbal medicine* Phytomedicines are currently the main precursors of HM-CDs. In addition, protein-rich medicines from animal drugs can act as sources of abundant heteroatoms or functional groups, which may help improve properties of HM-CDs. Therefore, CDs derived from animal drugs are a crucial area for future exploration.

*Reductions of toxicity and increasing efficiency* The minimum toxicity of HM-CDs triggered our consideration: Can HM-CDs achieve the purpose of reducing toxicity and increasing efficiency of toxic herbal medicine (such as manchurian dutchmanspipe stem, common monkshood, aristolochic acid)? If toxic reduction can be achieved, will the active ingredients change in the synthesis procedure, losing the original efficacy? Assuming it is feasible, to make toxic herbal medicine into CDs should be a novel processing tool. The solution to this issue will immensely drive the clinical applications of toxic herbal medicine.

*Recycle of herbal residues* The extraction efficiency of herbal medicine is approximately 50% [160]. The residual herbal residues also contain some vital active ingredients. Strategies to recycle herbal residues are underway. In the future, we must attempt to extract CDs from herbal residues, and reuse them in the diagnosis and treatment of diseases, achieving optimal utilization of herbal medicine.

## Data Availability

Not applicable.

## Abbreviations

HM-CDs: Herbal medicine-carbon dots
CDs: Carbon dots
JSX: Jiaosanxian
H-N-CD: Nitrogen-doped CDs from ginkgo fruits
QY: Quantum yield
BBB: Blood–brain barrier
SHC-CDs: Schizonepetae Herba Carbonisata-CDs
H-CDs: Ginkgo fruits-CDs using hydrothermal methods
M-CDs: Ginkgo fruits-CDs using microwave methods
E-CDs: Ethanol-papaya CDs
W-CDs: Water-papaya CDs
PTC-CDs: *Pollen Typhae Carbonisata*-CDs
APTT: Activated partial thromboplastin time
FIB: Fibrinogen
JMC-CDs: *Junci Medulla Carbonisata*-CDs
EYO-CDs: Egg yolk oil-CDs
PCC-CDs: *Phellodendri Cortex Carbonisatus*-carbon dots
MSC-CDs: Mulberry silkworm cocoon-derived carbon dots
LJFC-CDs: *Lonicerae japonicae* *Flos*-derived carbon dots
AFIC-CDs: *Aurantii fructus* *immaturus* *carbonisata*-derived carbon dots
RSFC: *Radix Sophorae Flavescentis* carbonisata
PLR-CDs: *Puerariae lobatae* *Radix* CDs
XOD: Xanthine oxidase
PRAC-CDs: *Paeoniae Radix Alba Carbonisata*-derived carbon dots
ALT: Alanine transaminase
AST: Acetone transaminase
TBA: Total bile acid
TBIL: Total bilium
MDA: Malondialdehyde
SOD: Superoxide dismutase
SCR: Serum creatinine
BUN: Blood urea nitrogen
UTP: Urine total protein
MALB: Microalbuminuria
AAFC-CDs: *Artemisiae Argyi Folium* Carbonisata-carbon dots
ZR: Zingiberis Rhizoma
ENK: Enkephalin
5-HT: Serotonin
IFE: Inner filter effect
FRET: Fluorescence resonance energy transfer
DL: Detection limit
LF-CDs: Lycii Fructus-CDs
PTN-CQDs: Nitrogen-doped Pinellia quantum dots
Pn-CDs: *Panax notoginseng* carbon dots
FAM: Carboxyfluorescein
6-MP: 6-Mercaptopurine
N/S-CDs: Nitrogen and sulfur co-doped carbon dots
y-CDs: Smilax China-derived yellow-fluorescent CDs
BPEI-CDs: Branched polyethyleneimine-CDs
LS-CDs: CDs derived from lychee seeds
MB: Methylene blue
SASP: Salazosulfapyridine
